# Measuring biological age in mice using differential mass spectrometry

**DOI:** 10.18632/aging.101810

**Published:** 2019-02-11

**Authors:** Harris Bell-Temin, Matthew J. Yousefzadeh, Andrey Bondarenko, Ellen Quarles, Jacqueline Jones-Laughner, Paul D. Robbins, Warren Ladiges, Laura J. Niedernhofer, Nathan A. Yates

**Affiliations:** 1 Department of Cell Biology, University of Pittsburgh School of Medicine, Pittsburgh, PA 15261, USA; 2 Department of Molecular Medicine, The Scripps Research Institute, Florida , Jupiter, FL 33458, USA; 3 Department of Biochemistry, Molecular Biology and Biophysics, and the Institute on the Biology of Aging and Metabolism, University of Minnesota, Minneapolis, MN 55455, USA; 4 Infoclinika, Bellevue, WA 98006, USA; 5 Department of Pathology, University of Washington, Seattle, WA 98195, USA; 6 Biomedical Mass Spectrometry Center, University of Pittsburgh Schools of the Health Sciences, Pittsburgh, PA 15261, USA; 7 Department of Comparative Medicine, School of Medicine, University of Washington, Seattle, WA 98195, USA

**Keywords:** proteomics, non-targeted, surrogate markers, aging, mouse, liver, mass spectrometry

## Abstract

Aging is an ill-defined process that increases the risk of morbidity and mortality. Aging is also heterogeneous meaning that biological and chronological age can differ. Here, we used unbiased differential mass spectrometry to quantify thousands of proteins in mouse liver and select those that that consistently change in expression as mice age. A panel of 14 proteins from inbred C57BL/6 mice was used to equate chronological and biological age in this reference population, against which other mice could be compared. This “biological age calculator” identified two strains of f1 hybrid mice as biologically younger than inbred mice and progeroid mice as being biologically older. In an independent validation experiment, the calculator identified mice treated with rapamycin, known to extend lifespan of mice, as 18% younger than mice fed a placebo diet. This demonstrates that it is possible to measure subtle changes in biologic age in mammals using a proteomics approach.

## Introduction

Aging is the greatest risk factor for the majority of chronic diseases. The world’s population is rapidly aging with the number of individuals over 65 estimated to double by 2050 [1]. More than 90% of adults over 65 have at least one chronic disease, and over two-thirds have two or more, accounting for >70% of deaths in America and 95% of all healthcare costs for the elderly [2]. Recently, efforts were initiated to begin therapeutically targeting aging with the goal of simultaneously delaying the onset of multiple chronic diseases [3,4]. The development of new treatments for aging will depend greatly on the identification of biomarkers that act as surrogates for measuring lifespan and healthspan, which are costly and lengthy to measure in pre-clinical models, let alone in humans [5].

Several hurdles complicate the discovery and development of molecular biomarkers of aging. This includes the time required to age animals, the need to test large numbers of candidate markers, and the translation of tests between models and species. Proteomic approaches offer attractive solutions to these challenges and hold great potential for identifying protein profiles that can serve as surrogate markers of “biological age”. First, mass-spectrometry based measures can examine thousands of candidate proteins from archived tissues and bio-fluids, thus economically enabling discovery of new biomarkers. Second, proteomic measures facilitate analysis of numerous strains of mice, genetic models of aging and treatment groups relative to a reference population, to accelerate testing and validation of biomarkers. Finally, mass spectrometry assays are based on the chemical measurement of specific amino acid sequences, not biological antibody recognition, thus allowing proteins to be tested across species with absolute molecular specificity.

Aging in most species is tremendously heterogeneous at both the organismal and molecular level [6,7]. As a result, precise measurements and sufficiently large sample sizes are needed to detect subtle, yet reproducible, changes in protein expression that occur as organisms age. In fact, a previous study using unbiased proteomics to detect global protein expression changes in mouse tissues that occur with aging yielded only 5 proteins, using a cut-off of 2-fold change in expression [8]. In a similar study on rat heart, the greatest differential in age-related protein expression changes was 2-fold [9]. Hence approaches are needed that enable detection of more subtle changes in protein expression.

Here, we took an unbiased proteomics approach to measure differences in protein expression in the livers of mice with aging. We chose to focus on liver because of the technical challenges associated with the large dynamic range (~10^10^) of protein concentrations in plasma [10]. One unique feature of our approach was that rather than a binary design (old vs. young), we analyzed at least 4 age groups over the lifespan of mice to identify changes in protein expression that occur with aging. A second unique feature of our approach was the analysis of a large number of samples (n=7-8 mice per age, sex, strain), which is only possible when using a label-free method of protein detection. The third unique feature was to utilize a differential mass spectrometry (dMS) workflow that prioritizes global protein quantification over identification (see [11–13] and Supplementary Figure 1 for details). This yields a molecular profile with hundreds of thousands of signals per liver that can be analyzed for age-related signatures and readily identified by amino acid sequence in follow-up analyses. The large sample size, multifactorial experimental design and emphasis on precise quantification greatly enhanced our ability to identify robust molecular features that exhibit a statistically significant yet subtle difference in expression between age groups. Finally, in a separate experiment performed nine months after the initial discovery study and utilizing liver samples obtained from an independent investigator/institution, we confirmed that the aging protein expression profile could be reproduced and used to detect a reduction in biological age induced by a drug intervention known to extend the lifespan of mice.

## RESULTS

**Figure 1:**
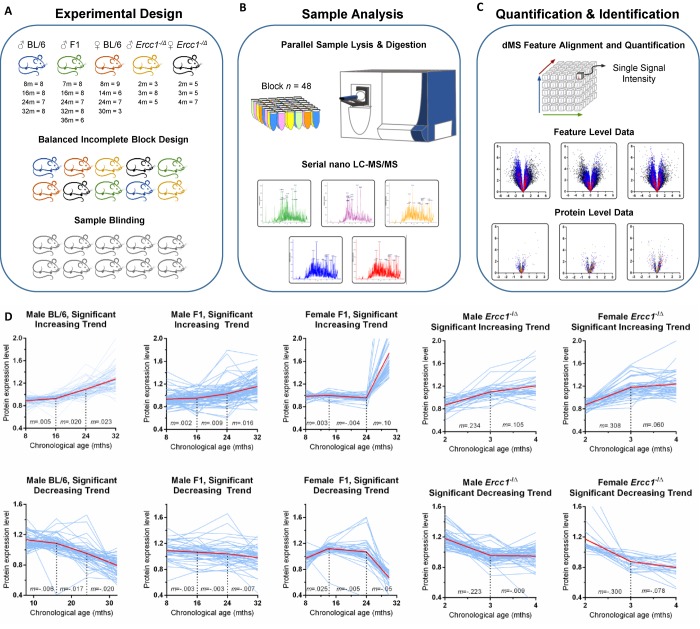
**Unbiased detection of age-related changes in protein expression in mouse liver.** (**A**) Details of input tissue samples (age in months and n per group) and methods of bias mitigation for sample preparation and analysis, including the creation of a balanced incomplete block design for all processing and analysis steps and sample blinding. For the *Ercc1*^-/Δ^ mouse liver the n refers to mutant mice / littermate controls. (**B**) Sample processing block size and representative mass chromatograms generated from each sample. See methods section for more detail. (**C**) Alignment, extraction, and storage of mass spectral feature data from raw mass spectrometer output based on retention time and accurate mass, allowing for quantification of each proteomic signal across all samples, results of which are shown in example feature level volcano plots. The y-axis is the negative log of p-value; the x-axis is the log fold-change in protein abundance. All features associated with a protein are combined to calculate protein expression as shown in volcano plots indicating proteins (individual dots) that were significantly increased or decreased in expression in old vs. young mice and the extent of that change in expression. (**D**) Plots of the relative abundance of all proteins (individual blue lines) that change significantly with aging as identified by one-way ANOVA. Protein expression was measured cross-sectionally throughout the lifespan of inbred male C57BL/6Jnia, male f1a (C57BL6/Jnia:Balb/cBy), female f1b (C57BL/6J:FVB/NJ), male f1b (C57BL/6J:FVB/NJ) *Ercc1*^-/Δ^ , and female f1b (C57BL/6J:FVB/NJ) *Ercc1*^-/Δ^ mouse livers. The graphs are separated into proteins that increased in expression with chronological age (top) or decreased (bottom). The red line represents the mean protein abundance for significantly altered proteins in that group. *m*= the slope between time points. Significance cutoffs as delineated in Supplementary Table 2.

The experimental design is illustrated in Figure 1. Male C57BL/6Jnia (inbred) mice were used as the reference population and compared to male mice in an f1 hybrid background (C57BL/6Jnia:Balb/cBy; called f1a) and female mice in a second f1 background (C57BL/6J:FVB/NJ; called f1b) (Figure 1A). The identities of the liver samples were blinded and a balanced incomplete block design (see methods for details) was used to minimize the impact of order bias that can be introduced during sample processing and analysis. Error introduced during sample processing and analysis was determined from the analysis of technical replicate samples from a pooled control. Sample preparation was carried out in parallel using a block size of 48 samples and then individual samples were analyzed sequentially by nanoflow liquid chromatography high-resolution mass spectrometry (nLC-MS). This label-free dMS workflow supports the unbiased quantification of proteomic features over the large number of samples required for this multilevel analysis (Figure 1B). Supplementary Table 1 lists all samples processed simultaneously in the initial discovery experiment (n=140 liver samples).

A cloud-computing dMS analysis pipeline (Infoclinika, Bellevue, WA) that enables the analysis of large nLC-MS data sets was used to detect and quantify levels upwards of a hundred thousand high resolution features per liver lysate. A data-cube structure was used to store the full dataset and enhance computationally intensive feature alignment and quantification calculations. Each feature is defined by its mass-to-charge ratio (*m/z*) and retention time (*rt*). The feature intensity (*i*) provided a relative measure of protein expression that can be compared across samples. Features that exhibited large fluctuations in intensity were removed from the dataset using occupancy filtering (features that appeared in <4 mice per strain/sex/age group) and outlier removal criteria (features that were greater than one order of magnitude outside the group median intensity level). Volcano plots of statistically significant features showed a similar number of features with increased or decreased expression with age (Figure 1C). Features linked to a common protein sequence were combined to yield protein-level expression data. More details on data analysis are provided in Supplementary Figure 1. It is important to note that differences in protein expression (x-axis, Figure 1C) were subtle (<1-fold increase) for the majority of proteins, illustrating why previous proteomic studies that used fewer numbers of samples failed to detect these differences as statistically significant [8,14].

In inbred male mice, 64,657 features passed occupancy and outlier filtering, which led to a total of 34,817 features quantified and identified with high precision. Tandem mass spectrometry data was used to link these features to 7,962 peptides that are uniquely found in 1,298 protein sequences. Detailed numbers of features and proteins quantified for each strain of mice is provided in Supplementary Table 2. Supplementary Tables 3-5 list the proteins for each of the strains of WT mice that met the minimum significance cutoff of <5% false discovery based on iterative random sampling strategy [15]. Differences in protein expression between age groups of mice was small (<20%) for the majority of proteins.

Line plots of relative protein abundance as a function of chronological age are shown for proteins that exhibited a significant increase or decrease with age (Figure 1D). Interestingly, the age when the most dramatic inflection in expression levels occurred is identical for over- and under-expressed proteins for a given mouse strain. But the age at greatest inflection in protein expression differed substantially between strains of mice. For example, in inbred male mice, protein expression is stable from 8-16 months of age then changes more dramatically between 16-24 and 24-32 months of age. In the longer-lived f1a male and f1b female mice, protein expression was stable into the third age group (24 months of age). After that (from 24 to 32 months), changes in protein abundance were more dramatic. This was particularly true in female mice, which is consistent with data indicating that several measures of health (body weight, percent fat mass and grip strength) drop more precipitously in female mice towards the end of life, whereas male mice experience a more steady decline in the last 12-16 months of life [16]. By analogy, women have a longer lifespan than men yet have greater disability and poorer health in old age [17].

Although not the main focus of this work, pathway enrichment mining was performed using the proteomic data from the oldest and youngest age groups for the three strains of wild-type mice (Supplementary Table 6). There was remarkable consistency in age-related protein expression levels between the three strains (inbred, f1a and f1b) of WT mice, even at the level of individual proteins. The pathways most significantly altered were oxidative damage/antioxidant response, fatty acid oxidation, nuclear receptors and clathrin-mediated endocytosis. Expression of proteins required for fatty acid oxidation was significantly reduced in liver of older mice compared to the younger age groups (Supplementary Figure 2 and 3) consistent with prior studies [18] and evidence that aged WT mice have fatty liver compared to younger adult animals (Supplementary Figure 2B). In contrast, expression of proteins related to endocytosis and phagocytosis were significantly increased with aging (Supplementary Figures 4 and 5).

**Figure 2:**
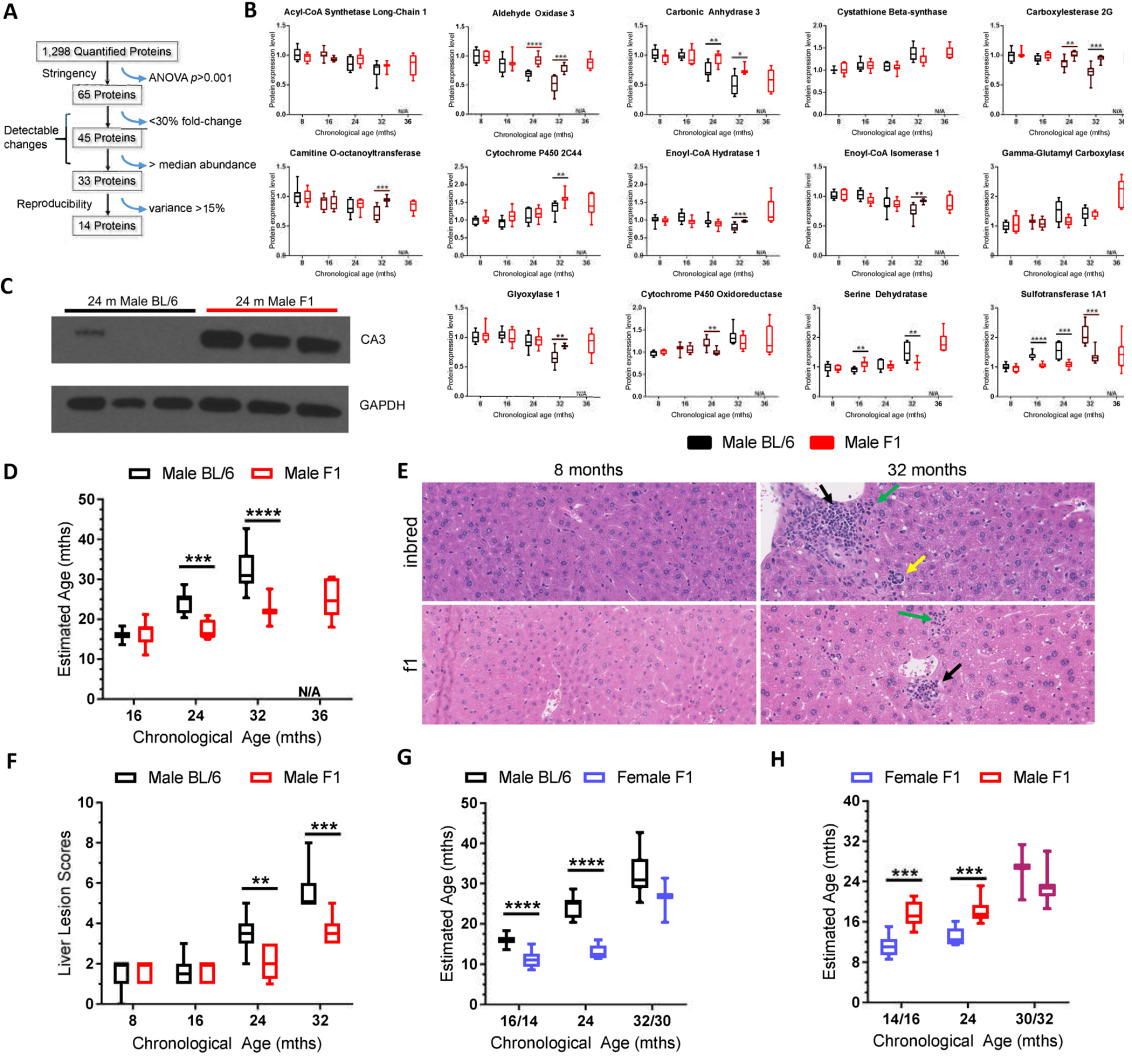
**Selection of the biological age calculator protein panel and its application to other strains of wild-type mice.** (**A**) Illustration of how the 14 protein panel was selected from the 1,298 proteins that were quantified in liver of C57BL/6Jnia mice. 65 proteins had a one-way ANOVA *p*<0.001, providing high statistical significance in age-related changes in expression. 45 of those proteins had a fold-change difference of ≥30% and 33 of those had abundance above the median, facilitating detection of expression changes. 14 of those 33 proteins had a maximal intragroup variance of 15%, supporting reproducibility. (**B**) Expression of the 14 proteins selected for the biological age calculator in male C57BL/6J mouse liver (black) and male f1a mouse liver (red) at multiple ages. (**C**) Immunoblot detection of carbonic anhydrase 3 expression in liver from three 24 month-old inbred and three f1a mice. The tissue lysates used were the same as those used in the MS experiments, providing intra-experimental validation. (**D**) The combined expression data from the panel of 14 proteins sets the biological (estimated) age to the chronological age for the reference group of mice, C57BL/6Jnia (i.e., the black bars define 16, 24 and 32 months according to the biological age calculator; see methods for more details). Red bars represent the summation of the data on the same 14 proteins in male f1a mice estimating their biological age at the chronological ages of 8, 16, 24, and 32 months relative to the reference strain. (**E**) Representative images of liver sections from male inbred (C57BL/6Jnia) and f1a (C57BL6/Jnia:Balb/cBy) mice at two ages. The older mice show numerous age-related lesions consisting of portal inflammation (green arrows), portal duct hyperplasia (yellow), microgranulomas (black) and mild intermittent hepatic degeneration. The inbred mice had more extensive age-related lesions than the f1a mice. (**F**) The composite lesion score reflects the incidence and severity of a specific panel of age-related liver lesions and was used as a separate calculator of biological age, in the same C57BL/6Jnia (black) and f1a (red) male mice used for proteomic analysis. (**G**) Same as (**D**) but for female mice of a different f1b strain (blue). The female mice were analyzed at 14 rather than 16 months of age and 30 vs. 32 months of age (x-axis). (**H**) Estimated biological age of f1b female (blue) vs. f1a male mice (red). Significance testing for all panels using Student’s unpaired, equal variance t-test, error bars show SEM. *p<0.05, **p<0.01, ***p<0.001, ****p< 0.0001; N/A, not applicable.

To determine if it is possible to define a signature of age-related changes from the unbiased data set, a subset of the 1,298 proteins quantified in inbred male mice were selected based on high sample occupancy, strong statistical significance, and low intra-individual variance (Figure 2A). The Jonckeere-Terstra ordinal trend test and the Kruskal-Wallis test were used to evaluate protein expression trends between samples (Supplementary Table 15) [19,20]. This yielded a panel of 14 proteins that were used to “set a biological clock” by which to gauge the aging of other mouse strains relative to C57BL/6Jnia inbred mice. A rudimentary linear model that weighted each of the 14 proteins equally was used to define the protein expression profile at 16, 24, and 32 months of age in a reference population of inbred mice (Supplementary Table 14). Thereby in C57BL/6 mice, chronological age was equated with biological age to create a frame of reference.

Expression of 11 of the 14 panel proteins differed between male f1a and male C57BL/6 mice, often at multiple time points, *i.e.*, multiple ages (Figure 2B). Notably, in all cases where there were differences between strains, if the expression declined with aging in inbred mice, this effect was blunted in the f1a mice, consistent with slower biological aging (Figure 2B). For example, the expression of carbonic anhydrase 3 (CA3) was observed to decrease dramatically with aging in C57BL/6 mice, but less dramatically in f1a mice (Figure 2B and Supplementary Table 3). Immunoblot detection of CA3 confirmed decreased expression in liver of 24 month-old inbred mice compared to age-matched f1a mice, consistent with the dMS data (Figure 2C).

Summation of the differences in expression of the 14 selected proteins enabled calculation of the biological age of f1a male mice relative to the inbred males (Figure 2D; see methods for details). We anticipated that the f1a male mice, which are known to be healthier and longer-lived [21–23] would have a younger “biological age” than chronologically age-matched inbred mice. Indeed, 24 month-old f1a mice appeared 16 months-old on the reference scale (*p*<0.001). 32 month-old f1a mice appeared 24 months-old (*p*<0.0001). Thus, by comparison to inbred mice, the f1 hybrid mice were calculated to be significantly younger biologically than their chronologic age.

These proteomic results were supported by pre-mortem functional data and post-mortem histopathologic analysis from the exact same mice as those used in the proteomics analysis. The f1a mice had significantly greater mean distance traveled in a voluntary running wheel at all ages, than did the inbred mice [24]. The f1a mice also maintained VO_2_ or energy expenditure into old age, unlike the inbred mice, which displayed a significant drop in VO_2_ in the eldest age group (Supplementary Table 7). These data are consistent with the f1a male mice being biologically younger than the inbred mice.

Post-mortem histopathologic analysis of liver revealed increased age-related histopathological lesions in the C57BL/6Jnia mice compared to that of age-matched f1a mice (Figure 2E). The composite lesion score (CLS) for a defined set of age-related liver lesions [25] was significantly lower in 24 and 32 month-old f1a mice compared to the inbred mice (Figure 2F). Analogous to the protein expression data (Figures 1D and 2B), the CLS for male C57BL/6Jnia mice was unchanged between 8 and 16 months, before increasing linearly between 16 and 32 months. In contrast, CLS for the male f1a mice was unchanged until 24 months of age. At 32 months, the f1a CLS was equal to that of the 24 month-old inbred mice. Thus, histopathologic analysis of liver is consistent with the proteomics data.

The total CLS derived from histopathologic analysis of four organs (heart, lung, kidney and liver) of the mice used for proteomics analysis was significantly lower in 24 and 32-month-old f1a mice compared to inbred mice (Supplementary Table 8), as were the organ-specific CLS for 3 of the 4 organs examined (Supplementary Table 9) supporting the conclusion that the two strains of mice age at different rates. This independent method of determining biological age was entirely consistent with the proteomics-based biological age calculator. Thus, both pre-mortem and post-mortem analysis of the mice used in the initial proteomics analysis indicate that the f1a mice are biologically younger than chronologically age-matched inbred mice, as the dMS proteomics analysis predicted.

**Figure 3:**
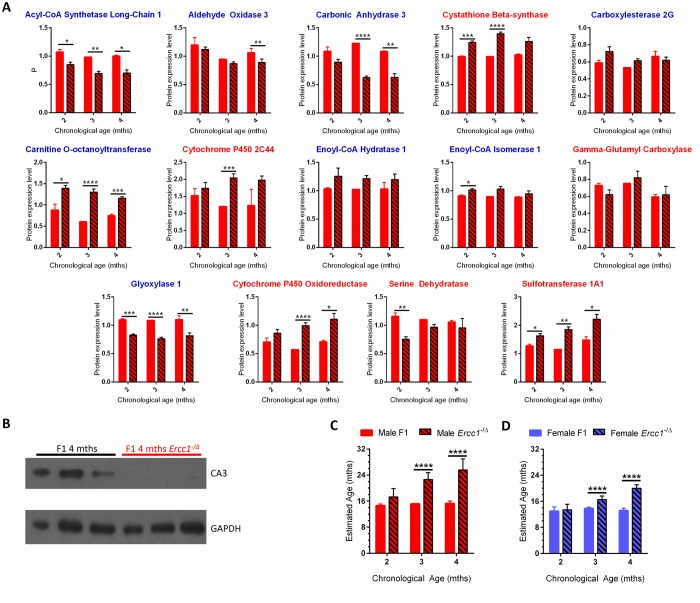
**Using the age calculator to determine the biological age of progeroid *Ercc1*^-/Δ^ mice.** (**A**) Expression of the 14 proteins selected for the biological age calculator in male f1 *Ercc1*^-/Δ^ mouse liver (hatched bars) compared to age-matched f1 male mice (solid red) at 3 ages. The proteins with blue titles decreased significantly in expression with chronological age of wild-type mice, while those with red titles increased significantly with aging in WT mice (Figure 2). Error bars show SEM. *p<0.05, **p<0.01, ***p<0.001, ****p< 0.0001. (**B**) Immunoblot validation of reduced expression of carbonic anhydrase 3 in liver of progeroid *Ercc1*^-/Δ^ mouse liver compared to wild-type littermates. Tissue samples were from mice distinct from the MS experiment, providing inter-experimental validation. (**C**) Estimated biological age of male f1 WT (red bars) and *Ercc1*^-/Δ^ (hatched bars) mice at three ages (x-axis) compared to the age of male inbred mice (y-axis). (**D**) Estimated biological age of female f1 WT (blue bars) and *Ercc1*^-/Δ^ (hatched bars) mice at three ages (x-axis) compared to the age of male inbred mice (y-axis). Significance testing for all panels using Student’s unpaired, equal variance t-test, error bars show SEM, ****p< 0.0001.

Based on the 14 signature proteins (Supplementary Figure 6), female mice in a distinct genetic background (called f1b) also were significantly biologically younger than the reference inbred male mice throughout the first two years of life (Figure 2G). At 14 and 24 months of age, the females were predicted to be 10 and 13 months-old, respectively. But by 30 months of age, their predicted biological age was roughly equivalent to their chronological age, consistent with the slopes of the trend plots shown in Figure 1D. This was recapitulated by comparing the female f1b and male f1a mice (Figure 2H), where female mice were calculated to be significantly younger than male mice through the first two years of life. But the sex-specific differences were absent by 30-32 months of age. This sex-specific differences in pace of aging is supported by the observation that expression of two markers of cellular senescence (*p16^Ink4a^* and *p21^Cip1^*), a well-recognized driver of aging [26,27], was significantly greater in liver tissue of male f1b than female f1b mice at 24 months of age, but the sex differences were all but lost by 32 months of age (Figure 3D).

The liver proteomics data from mice of both sexes and two genetic backgrounds compared to the reference inbred C57BL/6Jnia mice provides two examples where the profile of liver protein expression obtained by dMS predicted differences in the biological age between the groups of mice. This prediction was supported by our functional and histopathological data as well as prior studies indicating that f1 mice have a longer lifespan than inbred animals [21,22].

To further challenge the utility of the biological age calculator, we used genetic and pharmacologic approaches to accelerate and decelerate aging. *Ercc1*^-/Δ^ mice model a human progeroid syndrome [28], aging rapidly between 2-6 months of life [29]. Liver from 2, 3, and 4 month-old *Ercc1*^-/Δ^ mice was compared to age-matched WT mice, both in an f1b genetic background, using dMS. This yielded >1,000 proteins that were differentially expressed between the progeroid and WT mice (Supplementary Table 1, 2, 10 and 11). Plotting the expression of all of the proteins that were altered with aging in the *Ercc1*^-/Δ^ mouse liver revealed an inflection point at 3 months of age for both male and female mice (Figure 1D). The differences in protein expression between progeroid and WT mice rose more quickly between 2-3 months of age than between 3-4 months.

The expression level of 11 of the 14 panel proteins was significantly different between male *Ercc1*^-/Δ^ mice and age-matched WT congenic male mice (Figure 3A). For 8 of these 11 proteins, the differences in expression between *Ercc1*^-/Δ^ mice and age-matched WT mice was in the same direction as aging-related changes in WT mice, *i.e.,* if protein expression went down with aging in WT mice, it was lower in *Ercc1*^-/Δ^ mice compared to age-matched WT controls. Across the lifespan of the *Ercc1*^-/Δ^ mice, protein expression tended to trend in the same direction as what occurred with normal aging for all 14 proteins (at least from 2-3 months of age). Furthermore, as the mutant mice aged from 2-4 months, protein expression differed significantly from that in age-matched WT mice (*p*<0.001 for both sexes). Immunoblot detection confirmed decreased levels of CA3 in progeroid *Ercc1*^-/Δ^ mice compared to age-matched WT mice (Figure 3B).

Using the composite expression profile of the 14 panel proteins in C57BL/6Jnia male mice as a reference, the biological ages of chronologically age-matched WT and *Ercc1*^-/Δ^ f1b mice were estimated (Figure 3C-D). The biological age of young WT mice remained stable between 2-4 months of age, as expected. Their estimated age was between 8-16 months of age. This is because the youngest inbred mice used for dMS analysis were 8 months-old (Figure 1A). Furthermore, there were no significant differences in protein expression between 8 and 16 months of age in C57BL/6Jnia mouse liver (Figure 1D, Supplementary Table 3, Figure 2B). Thus, the lowest age that the biological age calculator can register is 8-16 months.

At 2 months of age, the *Ercc1*^-/Δ^ mice did not differ significantly in their calculated biological age from WT mice. However, the 3 month-old *Ercc1*^-/Δ^ mice were estimated to be significantly older than their WT counterparts having an estimated biological age of 23 months (*p<*0*.*0001). The 4 month-old *Ercc1*^-/Δ^ mice had an estimated biological age of 26 months. Similar trends were observed for female *Ercc1*^-/Δ^ mice (Supplementary Figure 8 and Figure 3D). The dramatic rise in the biological age of the progeroid mice between 2-3 months of age, with a subsequent flattening out in the rise from 3-4 months of age (Figure 3C-D) matches the trends in protein expression differences between age groups plotted in Figure 1D.

**Figure 4:**
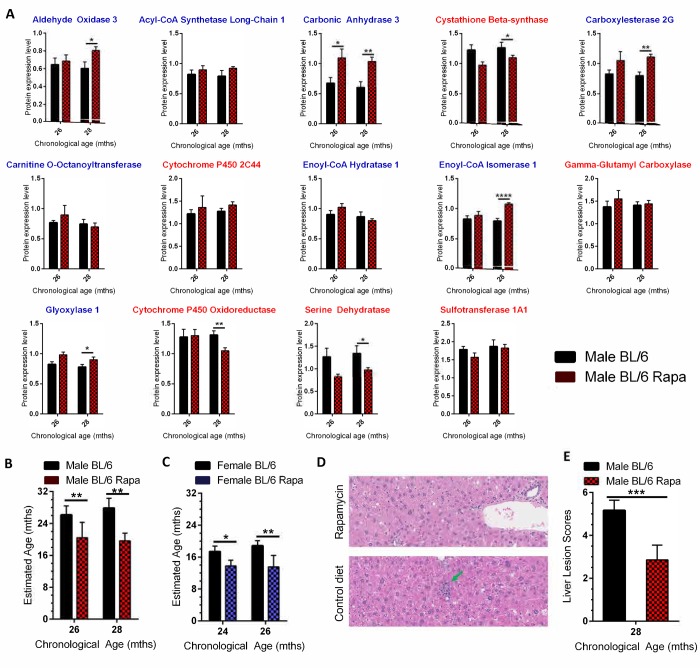
**Using the age calculator to determine the biological age of WT mice treated with the anti-geronic factor rapamycin.** (**A**) Expression of the 14 proteins selected for the biological age calculator in livers of C57BL/6NJ male mice put on a diet containing 42 ppm rapamycin starting at 24 months for 8 weeks (26 month data, red checked bars) or 16 weeks (28 month data, red checked bars) or mice fed a control diet (black bars). The proteins with blue titles above the graph decreased significantly in expression with age in C57BL/6NJ mice, while those with red titles increased significantly (Figure 2). (**B**) Estimated biological age for male C57BL/6NJ mice fed the rapamycin diet (red checked bars, 26 and 28 months represent 8 and 16 week treatment, respectively) relative to isogenic male mice fed a control diet (black bars). (**C**) Estimated biological age for female C57BL/6NJ mice fed the rapamycin diet (14 ppm; blue checked bars) relative to isogenic female mice fed a control diet (black bars) using C57BL/6NJ male mice as the reference (y-axis scale). (**D**) Representative images of liver sections from male C57BL/6NJ mice ± treatment with rapamycin. There was a lack of progression of age-related lesions (arrows) in the rapamycin-treated mice compared to mice on the control diet. Lesions consist of microgranuloma (green arrow) and mild intermittent hepatic degeneration. (**E**) The composite lesion score, reflecting the incidence and severity of a specific panel of age-related liver lesions in the rapamycin-treated male mice compared to isogenic mice on a control diet. Significance testing for all panels using Student’s unpaired, equal variance t-test, error bars show SEM. *p<0.05, **p<0.01, ***p<0.001, ****p< 0.0001.

Rapamycin extends the lifespan and healthspan of mice, slowing biological aging [30]. Since our goal was to define a molecular signature that predicts biological age as a surrogate marker of lifespan and age-related pathology that could be applied to interventional testing, we asked if the biological age calculator could detect the effects of rapamycin treatment. In addition, we wanted to confirm that the biological calculator would work on MS data collected in an independent experiment performed 9 months after the discovery experiment using biological specimens from an independent source. Analysis of 56 liver samples from C57BL/6NJ mice of both sexes fed a diet supplemented with rapamycin or a control diet (Supplementary Table 12) identified 1,641 that differed in expression between the groups (Supplementary Table 13). Measuring expression of the 14 proteins in the biological calculator revealed significant differences in expression of 8 proteins between treatment groups (Figure 4A).

In all cases, the rapamycin treatment reversed the aging trend (*i*.*e.*, increased the expression of proteins identified as declining in expression with aging). The biological age calculator was used to estimate the effect of rapamycin treatment on biological age of male and female mice (Figure 4B and 4C). Treating male mice for 8 weeks with rapamycin reduced their biological age from 26 to 20 months (*p*<0.01). Treating male mice with rapamycin for 16 weeks reduced their biological age from 28 months to 19 months. Treating female mice with rapamycin for 8 weeks slowed aging by ~2.5 months (Figure 4C). A 16 week treatment slowed female aging by ~5 months. The relative youthfulness of the liver from rapamycin-treated mice was confirmed by histopathological analysis (Figure 4D-E). These results validate the utility of our approach for measuring biological age in an independent experiment and demonstrates that the approach can be used to measure the effects of anti-aging interventions.

## DISCUSSION

There are remarkably few studies employing proteomics to identify age-related changes in protein expression. Nevertheless, such studies are desperately needed for discovery of novel biomarkers that correlate with aging and ideally frailty or biologic age. In addition, novel approaches are needed to establish that protein expression changes are reproducible between strains of mice, between experiments and between laboratories. To date, the majority of proteomics studies investigating aging have used a single strain of animal [8,31–33], with only one apparent exception [34]. The majority employed a binary comparison using just two age groups [8,31,32,34–36]. Most studies used four or less individuals per group [8,32,34–36] and many have yielded only a handful of proteins that are significantly differentially expressed in aged organisms compared to young [31,32,35]. None of the previous work that we are aware of reported two independent experiments in a single study to establish reproducibility of their analytical and statistical approaches.

Here, we addressed the need for novel proteomics analyses in the field of aging by taking a dramatically different approach, employing 3 genetic backgrounds of mice, 4-5 age groups, both sexes, and a larger number of mice per group. We report analysis of liver proteomics from 140 mice in the first experiment and 56 mice in the second. Analysis emphasized accurate quantitation of peptide levels in individuals, leading to the quantification of approximately 1,500 identified proteins across all samples, hundreds of which were significantly differentially expressed in each group (3 genetic backgrounds and both sexes) with aging. The expression level of fourteen of the most significantly and robustly altered proteins were used to create a composite measure of age using inbred C57BL/6Jnia mice as the reference strain. Use of this 14 protein panel identified two strains of f1 hybrid mice as being biologically younger than age-matched inbred mice. When biological age was altered genetically or pharmacologically, this was identified using the 14 protein composite expression analysis. Thus, our novel approach yielded a panel of proteins, not related to a single pathway or metabolic change that consistently change with aging across strains of mice and is responsive to a therapeutic intervention known to extend lifespan.

In conclusion, by using label-free dMS to analyze liver from a large cohort of mice, we discovered small reproducible differences in expression of a set of proteins that consistently predict the rate of aging, whether between different strains of mice, or if aging is accelerated or decelerated via genetic mutations or drug intervention. This establishes proof of principle that proteomic measurements are useful for defining markers of aging and that a small number of protein measurements can be used to define biological age. These molecular endpoints hold promise as surrogate biomarkers of longevity that could be used to assess the accuracy of genetically modified models of aging, but more importantly the efficacy of interventions that extend healthy aging. By translating our 14-proteins to a simple, targeted MS assay, it will be possible to develop a high-throughput molecular screen for the discovery and characterization of novel compounds that alter biological age. Importantly, this work further demonstrates unbiased dMS proteomics as a reliable method for the discovery of protein biomarkers and supports the extension of this work to additional tissues and biologically accessible fluids, including urine and plasma.

## MATERIALS AND METHODS

### Mice

Wild-type and *Ercc1*^−^*^/Δ^* mice in an f1 hybrid C57Bl/6J:FVB/NJ background were produced by crossing two inbred mice. These animals were generated and maintained at The Scripps Research Institute, Florida. Genomic DNA was isolated from ear tissue and the genotypes of the *Ercc1*^−^*^/Δ^* mice were determined by Transnetyx (Cordova, TN). Male C57BL/6Jnia and male f1 mice (C57BL/6Jnia:Balb/cBy) were obtained from the NIA Aged Rodent Resource and maintained at the University of Washington. These mice originated from The Jackson Laboratory. For the rapamycin study, which was conducted at the University of Washington, C57BL/6Jnia mice were obtained from the NIA Aged Rodent Resource. All mice were bred at Charles River Laboratory. The IACUC of The Scripps Research Institute, or the University of Washington at Seattle, approved all mouse studies.

### Proteomics analysis

The dMS analysis pipeline (Infoclinika, Bellevue WA) accepts multiple raw high-resolution mass spectrometry data files as an input and creates a datacube that holds mass spectral features that have been aligned and grouped over the entire dataset. These features are defined by their accurate mass/charge, retention time, and intensity and can be verified by manual comparison with the raw data using the instrument manufactures data analysis tools (QualBrowser). Feature intensity provides a relative measure of abundance and serves as the basis for quantification of protein expression. Quantification is performed by comparing the intensity of a feature across multiple samples and is carried out on hundreds of thousands of features per experiment. Noisy features were removed using occupancy and outlier filtering. Occupancy filtering removed features from the experiment appearing in less than half of all samples by group. Outlier filtering removed features per sample that were outside of a one order of magnitude range around the median intensity level. Stringency filtering removed noisy features from the analysis, improving quantification. Feature level quantifications were combined by protein to yield relative protein expression data.

A label-free differential mass spectrometry workflow was used to analyze high resolution LC-MS data for livers from 140 wild-type and progeroid mice from three genetic backgrounds and both genders (listed in Supplementary Table 1). Noise filtering was applied to each strain, gender and genotype separately to ensure that no data that was unique to a particular strain was removed. In addition, 56 samples of rapamycin-treated C57BL/6NJ mice of both genders and two lengths of treatment were analyzed in a separate experiment (listed in Supplementary Table 9).

### Sample preparation

In order to minimize bias in sample preparation and mass spectrometric analysis, samples were arranged in a balanced incomplete block design taking into account age, gender, strain, and genotype. The identities of the mouse liver samples were blinded until statistical analysis. Blinded samples were processed in batches of 48 samples. Approximately 100 mg portions of liver were dissected on ice and placed in 1 ml of 125 mM Tris-HCl, pH 7.6, and 100 mM dithiothreitol. Samples were lysed in a FastPrep-24 parallel homogenizer (MP Biomedicals, Santa Ana, CA) using lysing matrix D (MP Biomedicals, Santa Ana, CA) for 60 seconds at the 6.5 m/s setting 10% SDS was added in a 1:4 ratio for a final lysis buffer of 100 mM Tris-HCl, pH 7.6, 80 mM dithiothreitol, and 2% SDS. Samples were lysed at 99 °C for 5 minutes in a Thermomixer (Fisher Scientific, Waltham, MA) at 300 RPM. Samples were cooled to room temperature and insoluble materials removed by centrifugation at 22,500 RPM for 10 minutes.

Protein concentration was determined using a 660 nM Protein Assay with Ionic Detergent Compatibility Reagent (Fisher Scientific, Waltham, MA). 100 μg of protein was digested per sample using the filter-aided sample preparation (FASP) method as described by Wiṥniewski et al., with minor modifications [37]. Samples were added to 200 μl of 100 mM Tris-HCl, pH 8.0, 8M urea in an YM30 Microcon microcentrifuge filter (Millipore, Darmstadt, DEU). Samples were centrifuged for 15 minutes at 14,000 g before an additional 200 μl of urea buffer was added and centrifugation repeated. 100 μl of 100 mM Tris-HCl, pH 8.0, 20 mM iodoacetamide, 8M urea was added and samples incubated in the dark at room temperature for 20 minutes before centrifugation at settings above. Three 100 μl volumes of urea buffer were added and centrifuged between additions at 14,000 g for 14, 13, and 12 minutes, respectively. Three 100 μl volumes of 50 mM ammonium bicarbonate were added and centrifuged between additions for 12, 12, and 10 minutes, respectively. 100 μl of 50 mM ammonium bicarbonate with Sequence Grade TPCK-Treated Trypsin (Promega, Fitchburg, WI) were added to the samples in a ratio of 50 to 1 protein to trypsin by mass and the samples digested overnight in a humidified 37 °C incubator. Peptides were recovered into a new tube using two elutions of 100 μl of 50 mM ammonium bicarbonate and recovered via centrifugation at 14,000 x g for 10 and 15 minutes, respectively.

Samples were desalted on a vacuum manifold using 50 mg bed reversed-phase C18 solid phase columns (Supelco, Bellefonte, PA) as described previously [38]. Briefly, columns were activated with 0.1% formic acid in acetonitrile, followed by equilibration with 0.1% formic acid in water. Following loading, samples were washed with 0.1% formic acid in water. Samples were eluted in 90% acetonitrile, 1% water 0.1% formic acid. Samples were dried down in a Centrivap Concentrator with in-line cold trap (Labconco, Kansas City, MO) prior to resuspension in 0.1% formic acid in water at a concentration of 3 μg/μl.

### LC separation and mass spectrometric analysis

Samples were loaded in a NanoAcquity UPLC auto-sampler (Waters, Milford, MA) maintained at 4° C. A 1 μL aliquot of sample was directly injected using a flow rate of 300 nl/min onto a modular Picochip XL electrospray ionization chip (New Objective, Cambridge, MA) heated to 50 °C equipped with a 25 cm, 75 micron ID fused silica column filled with 1.9 μm reversed phased C-18 REPROSIL with 300 angstrom pore size (Dr. Maisch, DEU). Loaded samples were washed for 8 minutes in 3% acetonitrile in water and 0.1% formic acid prior to a 60 minute gradient to 32%. Flow was increased to 80% acetonitrile in 2 minutes and held for 8 minutes before a fifteen minute wash at 3% acetonitrile.

High resolution mass spectrometry data was acquired with a hybrid orbital ion trap mass spectrometer ORBItrap XL (Thermo Fischer Scientific, Waltham, MA). Full scan mass spectra were acquired with a resolution setting of 60,000. Tandem mass spectra were acquired using a data dependent acquisition method that collects 4 MS/MS spectra in the instruments linear ion trap mass analyzer. Automatic Gain Control target settings of 10^6^ and 5 x 10^3^ ions were used for full and dependent spectra, respectively, with a maximum fill time of 150 ms for dependent spectra. An exclusion list setting of 500 items was employed with a delay of 60 seconds. Data-dependent acquisitions settings were adjusted to exclude singly charged species and ions without an assigned charge state.

### Feature quantification and identification

All mass spectrometry data was uploaded to the publicly available data analysis suite Chorus (www.chorusproject.org). All proteomics data used in this manuscript will be deposited in a public project on the Chorus website upon publication of our manuscript.

A label-free differential mass spectrometry workflow was used for analysis and quantification of the high resolution LC-MS data. Native instrument files were translated from the vendor specific format (*.RAW) and converted to a vendor neutral format prior to analysis. Quantitative analysis aligned and quantified mass spectrometric signals, referred to here as “features”, as defined by their accurate mass/charge ratio, retention time, and relative intensity. Translated files are first processed as a two dimensional image with axes of accurate mass/charge and retention time, with the point size defined by relative intensity. Features are then clustered into isotope groups. A datacube is created by aligning isotope groups by retention time across all sample images. MS/MS data was matched to the high resolution full scan precursor masses and searched using Comet against the Uniprot reference set for Mus musculus. Full scan mass tolerance was set to 20 ppm and 0.8 Daltons for MS/MS scans, and two missed cleavages were allowed.

Sample identities, but not protein or peptide identities, were unblinded following Chorus quantification. Filtering was employed to remove noisy data. Samples were filtered using an occupancy filter requiring signal in at least half of all samples in a group, defined by age, strain and gender. Outlier filtering removed signals that were outside of an order of magnitude range from the median for a given group (age, strain and genotype of mice). Occupancy filtering was reapplied post outlier filtering.

Targeted identification was used to sequence significant features that were not selected for MS/MS spectra during the initial analysis. A Student’s unpaired, equal variance t-test was employed between the oldest and youngest age groups by strain and gender to detect significant unsequenced features. Unidentified features found to be significant in more than one gender or strain were selected for identification during additional scans over a two minute period around the signals retention time using a maximum fill time of 300 ms. Targeted identifications were added to the initial identification, and median normalized feature intensity by sample was assigned as protein intensity, using a minimum of 5 features from 2 peptides per protein.

dMS analysis of the naturally aged and progeroid mice, prior to occupancy and outlier filtering, identified 1,489 proteins deriving from 9,849 peptides and 585,332 mass spectrometric signals, or features during the initial identification stage. Analysis of the 28 liver samples from rapamycin treated mice and their age- and gender-matched controls (n=28) identified 1,641 proteins from 10,569 peptides and 522,259 features. 3,103 proteins and 14,083 peptides were identified across all experiments.

For the C57BL/6Jnia male mouse livers, across the ages of 8, 16, 24, and 32 months, 64,657 features passed occupancy and outlier filtering. Noise filtering have a pronounced effect on signals of lower intensity compared to those of higher intensity. In addition, high abundance signals have a greater chance of being selected for MS/MS analysis and result in the identification of an amino acid sequence. 1,146 unsequenced features, defined as features that were not selected for MS/MS analysis by data dependent acquisition, were found to be significant between the 8 month and 32 month groups (*p*<0.01). Targeted data acquisition was used to acquire MS/MS spectra for 408 of the un-sequenced features resulting in the identification of high confidence peptide identifications for 158 of the features not linked to a protein identification were successfully identified. In total, 34,817 features were quantified with high precision by differential mass spectrometry and matched to 7,962 peptide sequences that are unique to 1,298 proteins.

For the male C57BL/6Jnia:Balb/cBy mouse livers, 60,653 features passed filtering; 34,301 features were identified from 7,931 peptides and 1,268 proteins (Supplementary Tables 2 and 4). dMS analysis of the female f1 livers, yielded 76,683 features, 35,434 of which were identified from 7,088 peptides and 1,196 proteins. Noise filtering of mass spectrometric analyses of male *Ercc1*^-/Δ^ mouse livers aged 2, 3, and 4 months led to 46,292 features, 28,647 of which were identified from 6,924 peptides and 1,181 proteins. Noise filtering of female *Ercc1*^-/Δ^ mouse livers aged 2, 3, and 4 months resulted in 49,004 features, 30,290 of which were identified from 7,362 peptides and 1,238 proteins.

To determine the significance of the differences in expression of individual proteins across the various ages, a one way ANOVA was employed following an occupancy screen at the protein level allowing a single missing value per age group. For the 8, 16, 24, and 32 month C57BL/6Jnia male mouse livers, 316 proteins were found to have a *p*<0.05, 127 proteins with a p <.01 (see Supplementary Table 3 for a list), 55 proteins with a *p*<0.001, and 23 proteins with a *p*<0.0001. For the 8, 16, 24, 32, and 36 month C57BL/6Jnia:Balb/cBy male mouse livers, 436 proteins were found to have a *p*<0.05, 222 proteins with a *p*<0.01, 92 proteins with a *p*<0.001 (see Supplementary Table 4 for a list), and 44 proteins with a p<0.0001. For the 7, 14, 24, and 30 month C57Bl/6N:FVB/NJ female mice livers, 562 proteins were found to have a p<0.05, 362 proteins with a *p*<0.01, 167 proteins with a *p*<0.001, and 74 proteins with a *p*<0.0001. For the 2, 3, and 4 month male *Ercc1*^-/Δ^ mouse livers, 266 proteins were found to have a *p*<0.05, 138 proteins with a *p*<0.01, 46 proteins with a *p*<0.001, and 19 proteins with a *p*<0.0001. In the 2, 3, and 4 month female *Ercc1*^-/Δ^ mouse livers, 173 proteins were found to have a *p*<0.05, 69 proteins with a *p*<0.01, 14 proteins with a *p*<0.001, and 3 proteins with a *p*<0.0001.

In order to correct for false positive errors, due to multiple hypothesis testing, an empirical iterative random resampling strategy was employed (see reference 15, Tusher VG, Tibshirani R. and Chu G.). False positives are incorrect assignment of statistical significance due to the number of statistical analyses attempted. False discovery of randomly significant ANOVA analysis per protein was limited to less than 5% of true discovery in the male C57BL/6NJnia, male f1, female f1, male *Ercc1*^-/Δ^ f1, and female f1 *Ercc1*^-/Δ^ mice at an ANOVA value of p<0.01, p<0.001, p<0.0001, p<0.001, and p<0.01, respectively. Groups with the fewest number of samples have a greater likelihood of false positives due to random chance, as well as groups with the least linear differences between age groups.

To find if trends were similar between and within the sample groups, analysis of the linear relationship between ages was performed. Trends between age groups were evaluated by taking the square of the Pearson product moment coefficient of the median expression level per age. Of the 127 significant proteins for the male C57BL/6NJnia livers, 39 proteins showed an R^2^ value of >0.95 for the entire age range of 8 to 32 months, 49 proteins had an R^2^ value of >0.95 between 8 and 24 months, and 56 demonstrated significance across the age range of 16 – 32 months. Analysis of the significant proteins for C57BL/6Jnia:Balb/cBy mouse livers across the expanded age range of 8 to 36 months showed only a single protein with an R^2^ value >0.95, 7 proteins from 16 vs. 36 months, 9 proteins from 8 vs. 32 months, but 26 proteins between 24 and 36 months. Significant expression level differences for the male f1 livers appeared to occur later in life than for the inbred male mice.

For the female f1 mouse livers, trend analysis showed greater concurrence between the 7, 14, and 24 month old groups than the 14, 24, and 30 month groups. For the proteins determined significant by ANOVA analysis, only 13 proteins had a Pearson product moment coefficient of >0.95 for the entire age range. 15 proteins had an R^2^ >0.95 between 14 and 30 months. 50 significant proteins, however, had an R^2^ of >0.95 for the age range from 6–24 months. 24 out of 74 significant proteins from the female f1 wild-type data set demonstrated a significant reversal in direction between 6 and 24 months compared to 14 to 30 months. In addition, the median rate of change for significant proteins is 500% greater between the 14 and 30 month old mice than between the 6 and 24 month-old mouse expression levels. In comparison, for the male C57BL/6Jnia:Balb/cBy mouse liver proteins, only 5 of the 127 significant proteins showed a significant reversal of trend across the various ages, and the median rate of change was 80% greater between the oldest three age groups compared to the youngest three age groups.

The female *Ercc1*^-/Δ^ mouse livers were from subjects of 2, 3, and 4 months of age. Trends for select significant proteins are shown in Supplementary Figure 6. Of the 69 proteins found significant via ANOVA analysis, 8 were highly significant, *p<*0*.*001, in the female f1 data set between the 6 and 30 month age groups and 7 out of 8 trended in the same direction in both data sets.

### Biological aging calculator

The proteins used for the biological age calculator were selected using data from the inbred C57BL/6Jnia male mice as a reference population. The following selection criteria were used: 1) one-way ANOVA analysis p<0.001 to provide rigor, 2) an average protein expression difference of ≥30% between the youngest and oldest age groups to address sensitivity issues in other detection formats, 3) protein abundance greater than the median abundance for the entire dataset to enhance detectability, 4) intragroup (by age) coefficient of variation no greater than 15%, to enhance reproducibility. 14 proteins passed the criteria to form the panel used as the biological age calculator. For each of the 14 proteins, the average protein expression was plotted (y-axis) vs. chronological age (x-axis) for C57BL/6Jnia male mice at each chronological age (8, 16, 24 and 32 months). Linear regression analysis was used to create a best-fit trend line to illustrate how the expression of each individual protein changed with chronological age in the reference population. The slope and intercept of the linear regression was calculated for each protein for 16 to 32 months. This created a reference by which to compare other sexes, strains and interventions to calculate their biological age relative to the reference population.

To apply the aging calculator to the other strains of wild-type and progeroid *Ercc1*^-/Δ^ mice (both male and female), the protein expression level for each of the panel proteins was entered into the linear regression model (y-value) and was used to predict a biological age (x-value) for each individual animal. The median biological age across all 14 proteins was calculated to create the biological age for that animal. All animals in that group (same sex, strain, genotype or intervention group) were then averaged to create a predicted biological age relative to the reference population. In the case of the rapamycin intervention study, analysis was performed as a separate experiment. Thus, it was necessary to first normalize protein expression measured between the first experiment containing the reference samples and the rapamycin experiment. This was done by adjusting the mean protein expression for each of the 14 panel proteins in the control group (mice in the second experiment that got the control diet with no rapamycin) to the mean protein expression in the reference population of the first experiment. This was deemed appropriate because the mice were of the exact same strain, sex and chronological age. This enabled determination of the effect of rapamycin on the biological age of mice.

### Pathway analysis

Pathway enrichment mining was performed using the proteomic data for the oldest and youngest age groups for each of the strains of wild-type mice (Supplementary Tables 3-5). Protein expression ratios (old vs. young) were input into the Ingenuity Pathway Analysis suite e (IPA; Qiagen, Valencia, CA) to identify overrepresented pathways. For all three wild-type strains of mice (both sexes), proteins involved in fatty acid β-oxidation decreased in expression with aging in liver. Downregulation of proteins with a gene ontology term for fatty acid β-oxidation have a *p<*0*.*001 (male inbred C57BL/6Jnia mice), *p<*0*.*01 (male f1 C57BL/6Jnia:Balb/cBy mice), and *p<*0*.*01 (female f1 C57BL/6J;FVB/NJ mice), respectively, calculated with a Student’s two-tailed, equal variance t-test (Supplementary Table 6, Supplementary Figures 2 and 3). Proteins involved in glutathione production also became significantly less abundant with aging in all three wild-type strains of mice with *p<*0*.*05 (male inbred C57BL/6Jnia mice), *p<0.*01 (male f1 C57BL/6Jnia:Balb/cBy mice), and *p<0.*05 (female f1 C57BL/6J;FVB/NJ mice), respectively (Supplementary Table 6). The rate limiting step of glutathione production, however, is governed by GCLC, the glutathione cysteine ligase catalytic subunit, which is downregulated in all three wild type strains, significantly so in inbred male and f1 female mice. Additionally, significant alterations were observed in protein expression levels s involved in immune responsive clathrin-mediated endocytosis (Supplementary Table 6, Supplementary Figures 4 and 5). Expression of proteins involved in clathrin-mediated endocytosis were significantly increased in all three strains of wild-type mice (*p<*0*.*0001 for all three strains).

### Rapamycin study

Two year-old WT C57BL/6NJ mice were obtained from the National Institute of Aging Charles River colony. Mice were housed at 20°C in an AAALAC accredited facility under Institutional Animal Care and Use Committee (IACUC) supervision. After a brief acclimation period, the mice were randomly assigned to groups and fed a diet with microencapsulated rapamycin (Rapamycin Holdings, San Antonio TX) at 2.24 mg/kg/day (or 14 ppm in diet for female mice) or 6.72 mg/kg/day (42 ppm in diet for male mice) or a control diet of the same composition (including the encapsulation material). The animals were fed the diets for 8 or 16 weeks. All treatment groups are summarized in Supplementary Table 9.

Male C57BL/6NJ mice were treated with rapamycin for 8 or 16 week time courses starting at 24 months of age. Proteomic analysis of these samples yielded 34,730 features following noise filtering, resulting in data for 1,641 proteins. Statistical analysis performed consisted of a Student’s unpaired equal variance two tailed t-test between control and treatment for each treatment length. For the 8 week treatment versus age-matched control, 7 proteins were significant with *p<*0*.*001. For the 16 week treatment versus age matched control, 18 proteins were significant with *p<*0*.*001 (Supplementary Table 10).

Female C57BL/6NJ mice were treated with rapamycin for 8 or 16 weeks starting at 22 months of age. Statistical analysis performed consisted of a Student’s unpaired equal variance two tailed t-test between control and treatment for each treatment length. 14 and 2 proteins were significant with a *p<*0*.*001 from the 8 week and 16 week treatment groups, respectively.

### Histological staining

Animals were euthanized by CO_2_ asphyxiation at the indicated ages. Tissues were excised and fixed overnight in 4% paraformaldehyde. Following progressive tissue dehydration with ethanol and xylene, the tissues were embedded in paraffin. Sections (4  μm thickness) were subjected to H&E staining. Alternatively, tissues were fixed frozen by placing them in 2% paraformaldehyde for 2-3 hrs followed by submersion in 30% sucrose for 24 hrs with several changes of solution. The fixed tissues were then embedded in OCT for cryosectioning (6 μm thickness) and staining with LipidTox (ThermoFisher) to detect fatty infiltration.

### Histopathological scoring

Lesion scoring used the Geropathology Grading Platform (GGP) developed by the Geropathology Grading Committee [25]. The GGP is based on a standard set of guidelines designed to 1) detect the histological presence or absence of low impact lesions in multiple organs; and 2) measure the level of severity of high impact lesions related to aging in mice. The platform generates a numerical score for each lesion in a specific organ, so that a total lesion score is obtained by adding each lesion score for that organ for one mouse. Total lesion scores are averaged between all mice in a specific cohort to obtain a composite lesion score (CLS) for that organ. The CLS can then be used to compare response to drug treatment over time, determine effect of alterations in gene expression, or investigate the impact of environmental challenges in a variety of preclinical aging studies.

### Immunoblotting

Livers were homogenized in RIPA buffer using a FastPrep-24 homogenizer and incubated on ice for 30 min. Samples were centrifuged at 17,000 x g for 15 min at 4 ^o^C. Supernatants were resuspended in 2X SDS loading buffer and 25 or 100 µg of total protein was run on a 4-15% SDS-PAGE gel before being transferred to nitrocellulose membrane. Membranes were blocked for 1 hr in 10% milk TBS-T solution at room temperature before overnight incubation in anti-CA3 (Abcam, Cambridge, MA, catalog# ab181358, 1:500-1000) and anti-GAPDH (Abcam, catalog# ab8425, 1:5000) antibody in TBS-T at 4 ^o^C. After washing, samples were incubated in goat anti-rabbit HRP secondary antibody (Thermo-Fisher, catalog # 656120, 1:2000) in 5% milk TBS-T solution for 3 hr before washing and visualization with ECL (Thermo-Fisher).

## Supplementary Material

Supplementary Figures

Supplementary Tables
