# Supplementary material for: Measuring biological age in mice using differential mass spectrometry
Source: Aging (Albany NY). 2019 Feb 11;11(3):1045–61. doi: 10.18632/aging.101810 (PMC6382423; doi:10.18632/aging.101810)
Supplement: Supplementary Figures [file aging-11-101810-s001.pdf]

SUPPLEMENTARY FIGURES

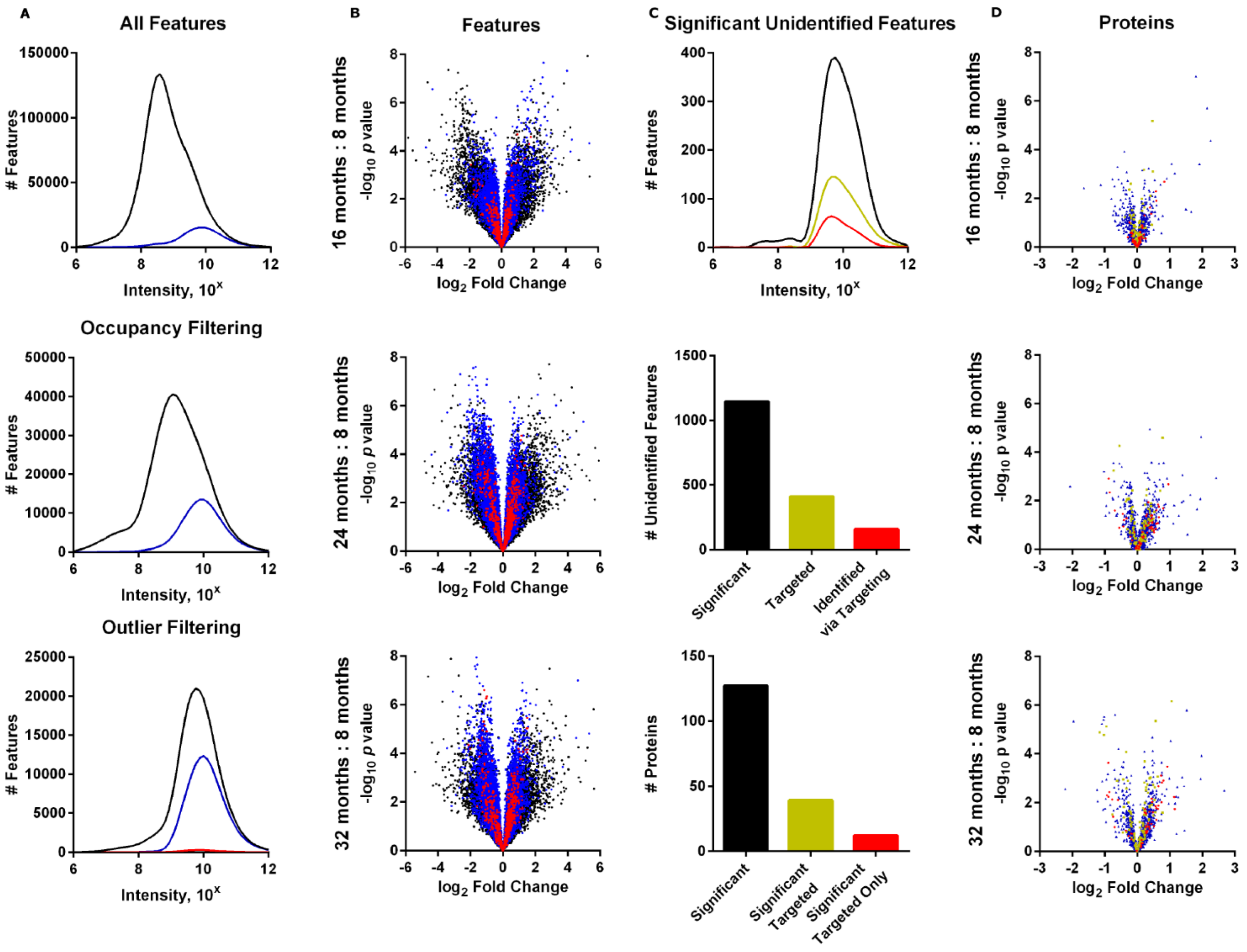

Supplementary Figure 1. Differential Mass Spectrometry Processing Results. (A) Effect of occupancy and outlier filtering upon intensity distribution of identified (blue) and non-identified (black) features. As filters are applied, the number of unidentified features decreases while the number of identified features remain stable, due to their higher intensity. **(B)** Volcano plots of feature abundance for male C57BL/6Jnia mice at 16, 24, and 32 months versus 8 months for unidentified features (black), first pass identified features (blue), and selectively identified proteins (red). **(C)** Intensity distribution of significant unidentified features (black), significant unidentified features selected for additional identification attempts (gold) and successful selective identifications (red). Of the 127 proteins that differed significantly between age groups of male C57BL/6Jnia mice, 60 included data from the additional identification runs, and 20 proteins were only identified due to the additional selective identification runs. Additional targeted analysis, possible only in a dMS workflow, added considerable data to significantly altered proteins. **(D)** Protein abundance for 16, 24, and 32 months versus 8 months, showing proteins that were identified solely through data-dependent analysis (blue), mixed data dependent/selective analysis (gold), and selective analysis (red).

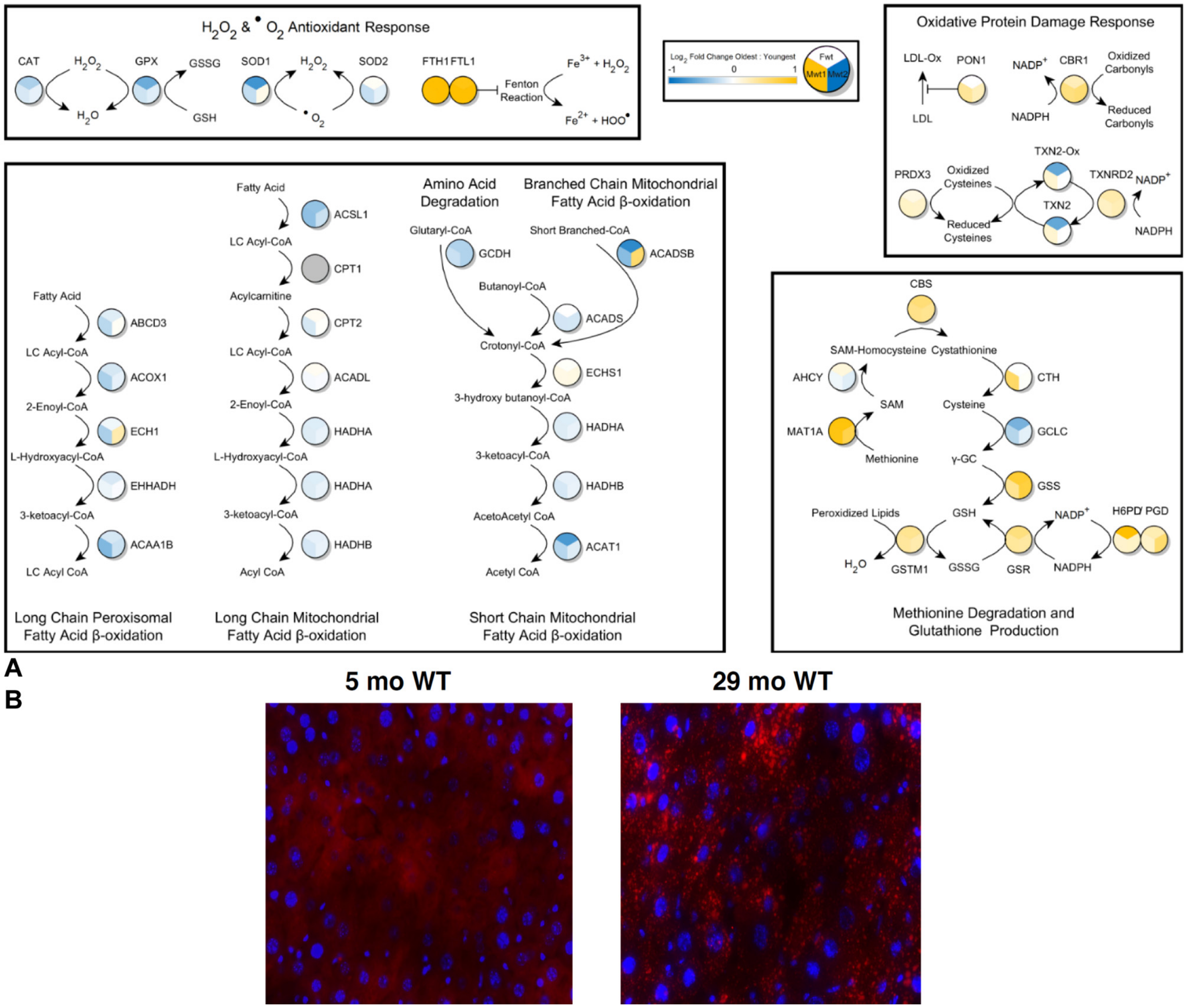

Supplementary Figure 2. Proteins involved in fatty acid β-oxidation and antioxidant pathways that differ significantly between young and old wild-type mice. (A) Indicated are the $\log_2$ of the ratio of protein expression in old vs. young mouse liver in three strains of wild-type mice. Gold is used to indicate proteins where expression was greater in old mice than young. Blue is used to indicate where protein expression was decreased in old mice compared to young. The darker the shade, the greater the difference in protein expression between young and old mice. Numeric ratios are found in Supplementary Tables 3-5. Trends between age groups for all proteins in the figure can be found in Supplementary Figure 3. Downregulation of proteins with a gene ontology term for fatty acid β-oxidation have a $p<0.001$ (male inbred C57BL/6Jnia mice), $p<0.01$ (male f1a C57BL/6Jnia:Balb/cBy mice), and $p<0.01$ (female f1b C57BL/6J;FVB/NJ mice), respectively, calculated with a Student's two-tailed, equal variance t-test. Proteins involved in glutathione production were also significantly downregulated in old mice compared to young in all three wild-type strains with $p<0.05$ (male inbred C57BL/6Jnia mice), $p<0.01$ (male f1a C57BL/6Jnia:Balb/cBy mice), and $p<0.05$ (female f1b C57BL/6J;FVB/NJ mice), respectively. **(B)** Representative images of LipidTox staining of liver sections from young and old f1b mice illustrating steatosis in old animals. Fatty liver is a manifestation of reduced fatty acid metabolism. Abbreviations: **Fwt**- C57BL/6J:FVB/NJ, **Mwt1**- C57BL/6Jnia, **Mwt2**- C57BL6/Jnia:Balb/cBy.

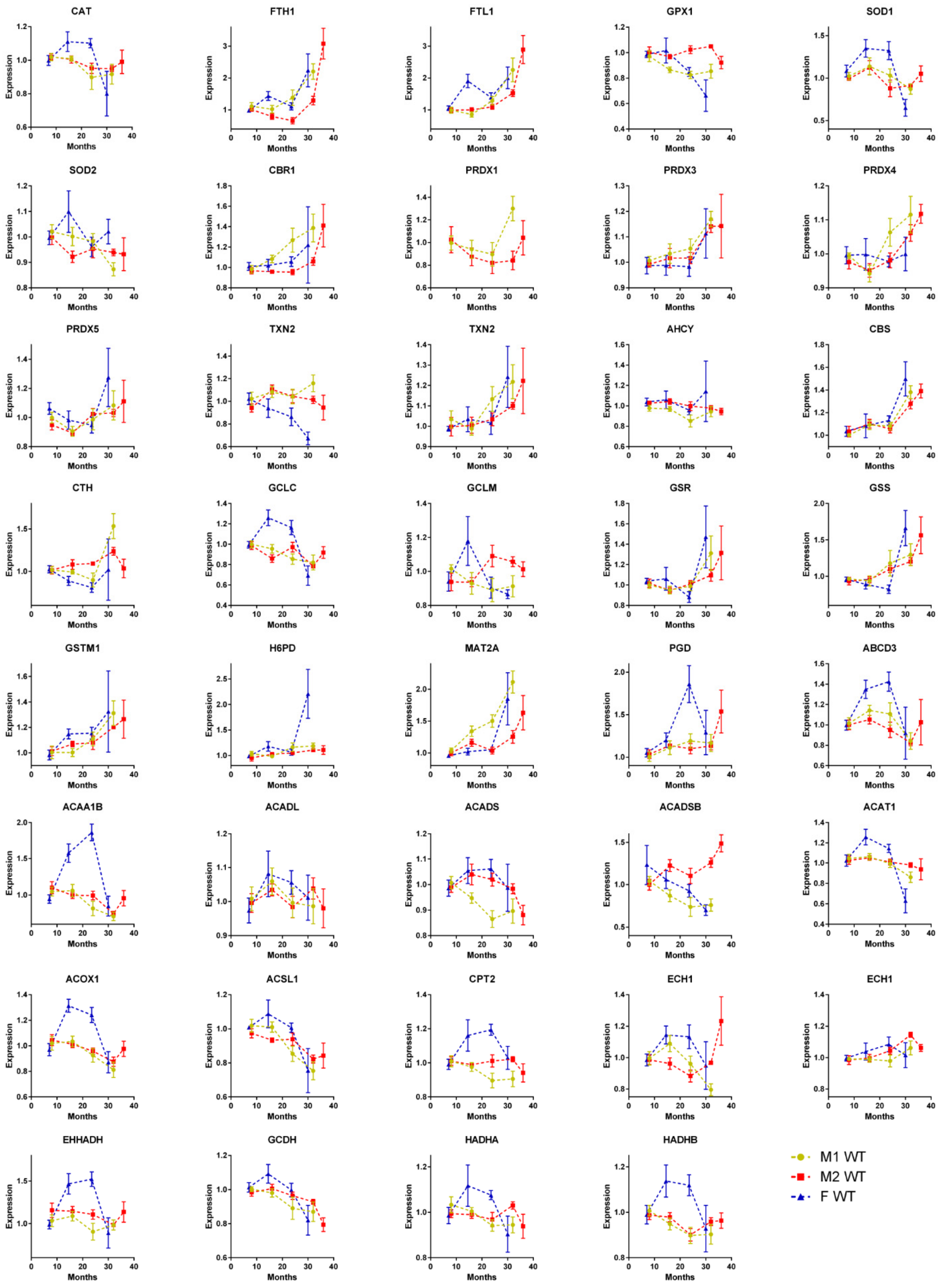

Supplementary Figure 3. Differences in protein expression with chronological age for proteins related to fatty acid β-oxidation and antioxidant pathways. Protein expression is plotted versus chronological age of male inbred C57BL/6Jnia (gold), male f1a C57BL6/Jnia:Balb/cBy (red), and female f1b C57BL/6J:FVB/NJ (blue) mouse liver for significantly altered proteins related to these pathways.

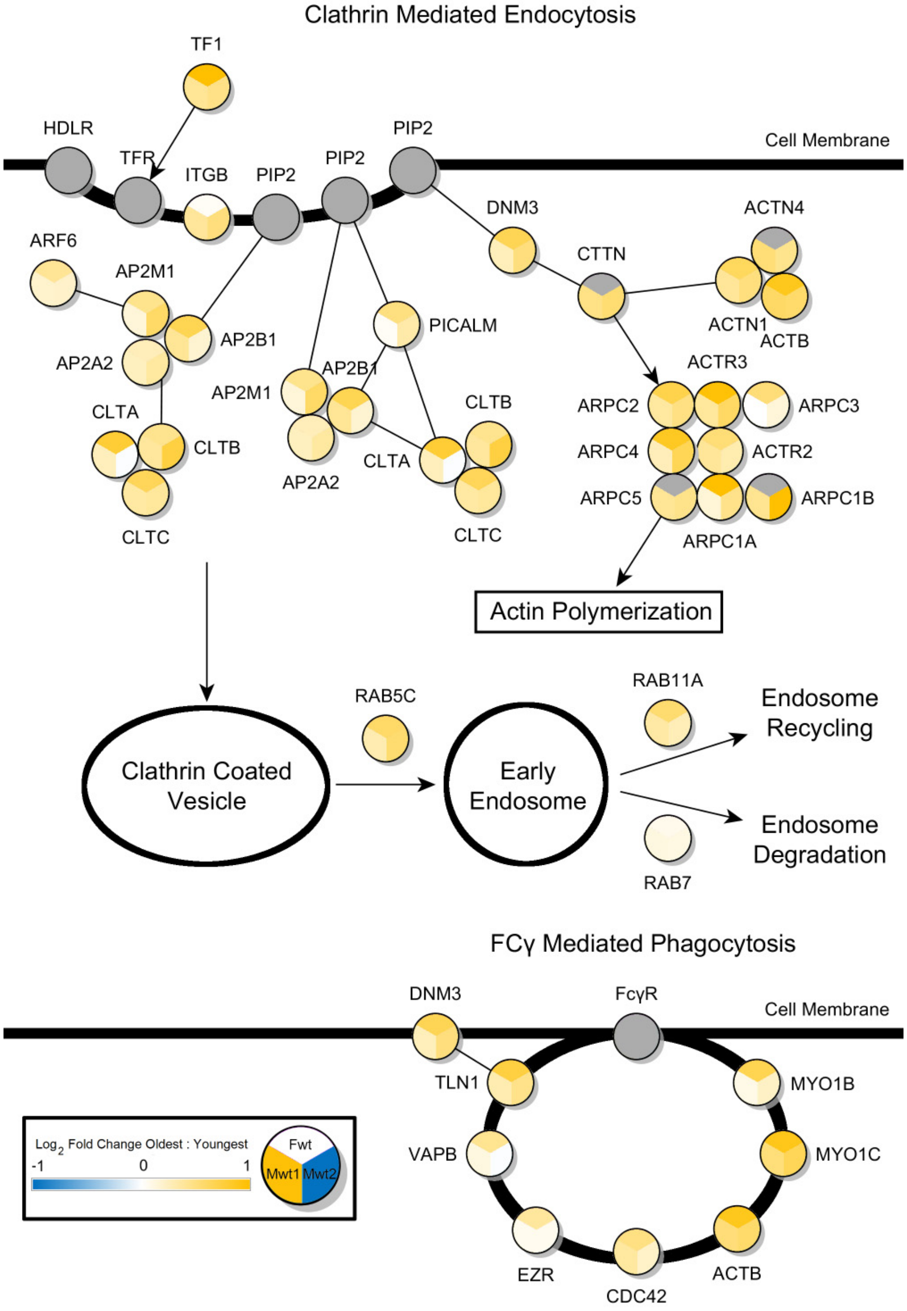

Supplementary Figure 4. Differences in protein expression with chronological age for proteins associated with clathrin-mediated endocytosis and phagocytosis. Indicated are the $\log_2$ of the ratio of protein expression in old vs. young mouse liver in three strains of wild-type mice. Gold is used to indicate proteins where expression was greater in old mice relative to young. The darker the shade, the greater the difference in protein expression in old vs. young mouse liver. Numeric ratios are found in Supplementary Tables 3-5. Expression of proteins involved in clathrin-mediated endocytosis were significantly increased in old mouse liver relative to young, in all three strains of wild-type mice ($p<0.0001$ for all three strains calculated with a Student's two-tailed, equal variance t-test). Trends between age groups for all proteins in the figure can be found in Supplementary Figure 5. Abbreviations: **Fwt-** C57BL/6J:FVB/NJ f1b, **Mwt1-** C57BL/6Jnia, **Mwt2-** C57BL6/Jnia:Balb/cBy f1a.

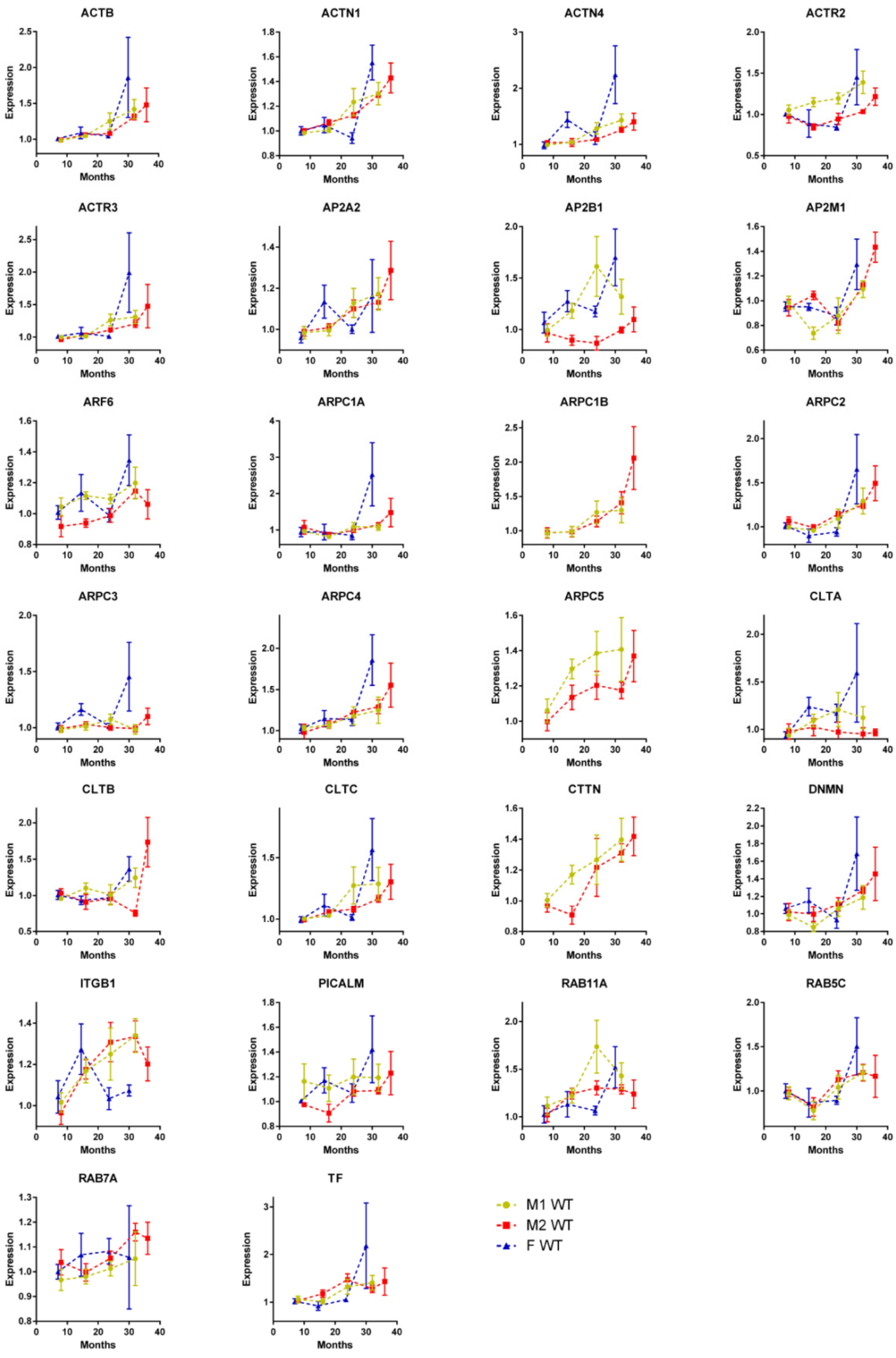

Supplementary Figure 5. Differences in protein expression with chronological age for proteins related to clathrin-mediated endocytosis and phagocytosis. Protein expression is plotted versus chronological age of male inbred C57BL/6Jnia (gold), male f1a C57BL6/Jnia:Balb/cBy (red), and female f1b C57BL/6J:FVB/NJ (blue) mouse liver for significantly altered proteins related to these pathways.

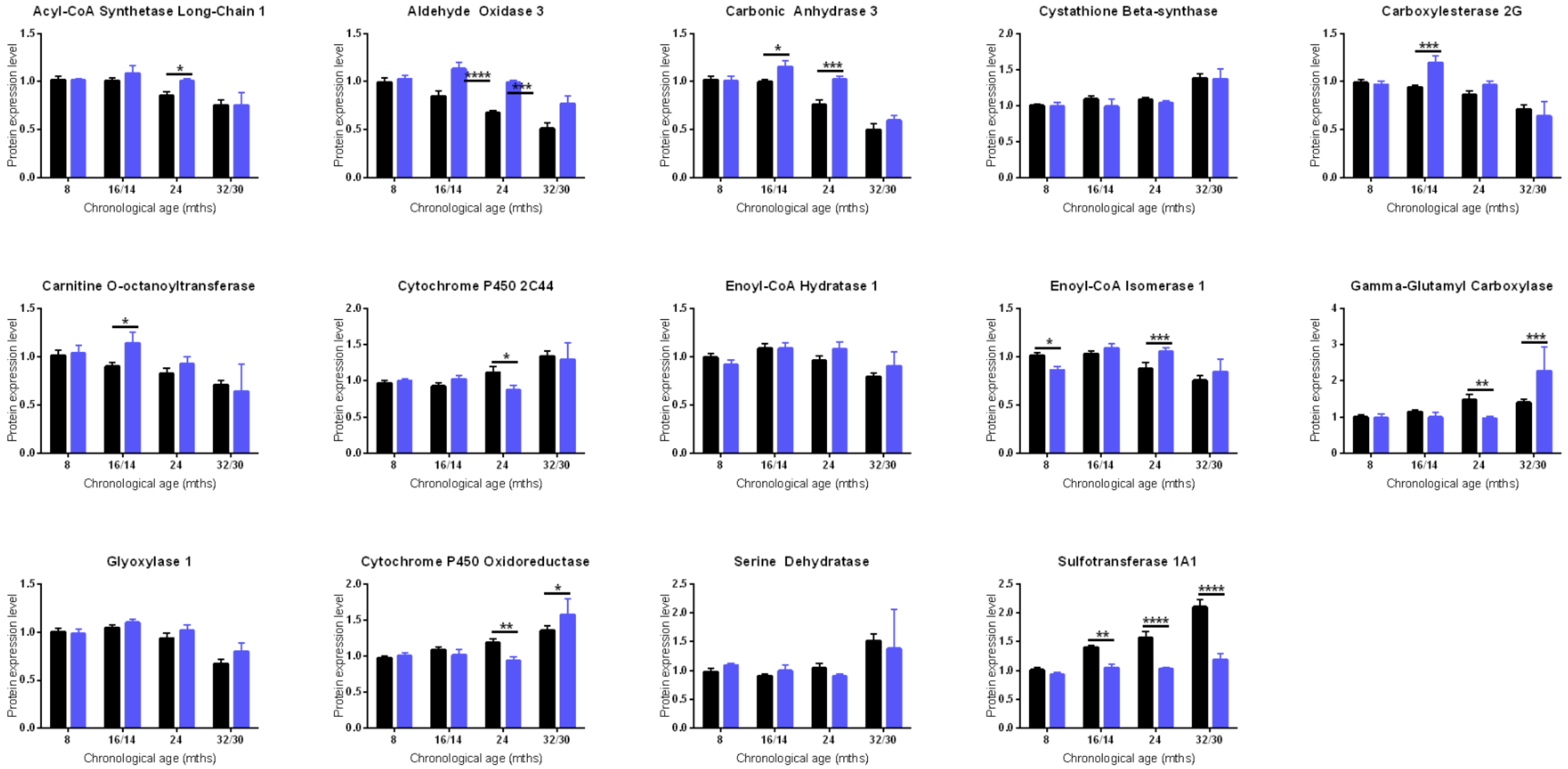

Supplementary Figure 6. Protein expression for the panel of 14 selected proteins in male inbred mouse liver vs. female f1b mouse liver. Expression of the proteins selected for the biological age calculator in male C57BL/6Jnia (black) and female f1b C57BL/6J:FVB/NJ (blue) mouse liver at multiple ages. Error bars indicate SEM. *p<0.05, **p<0.01, ***p<0.001, ****p< 0.0001 by one-way ANOVA.

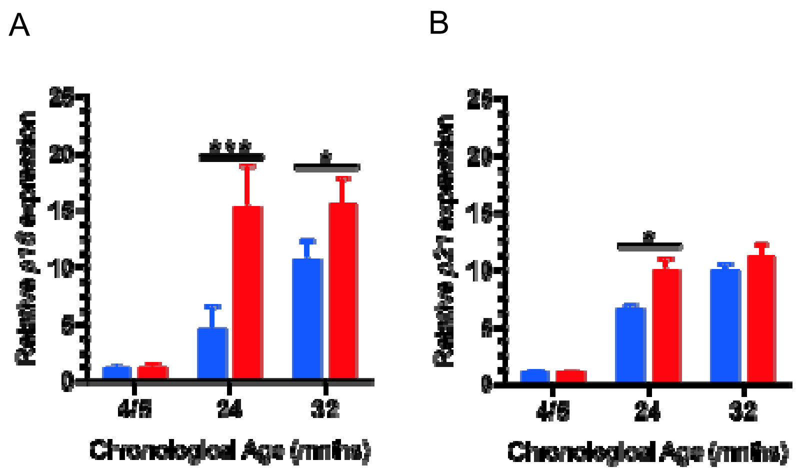

Supplementary Figure 7. Expression of two markers of cellular senescence in liver of male and female f1b WT C57BL/6J:FVB/NJ mice. (A) $p16^{Ink4a}$ and **(B)** $p21^{Cip1}$ mRNA were measured in liver from male (red) and female (blue) mice at multiple ages. Error bars indicate standard error. **p*<0.05, ****p*<0.001, unpaired two-tailed Student's *t* test.

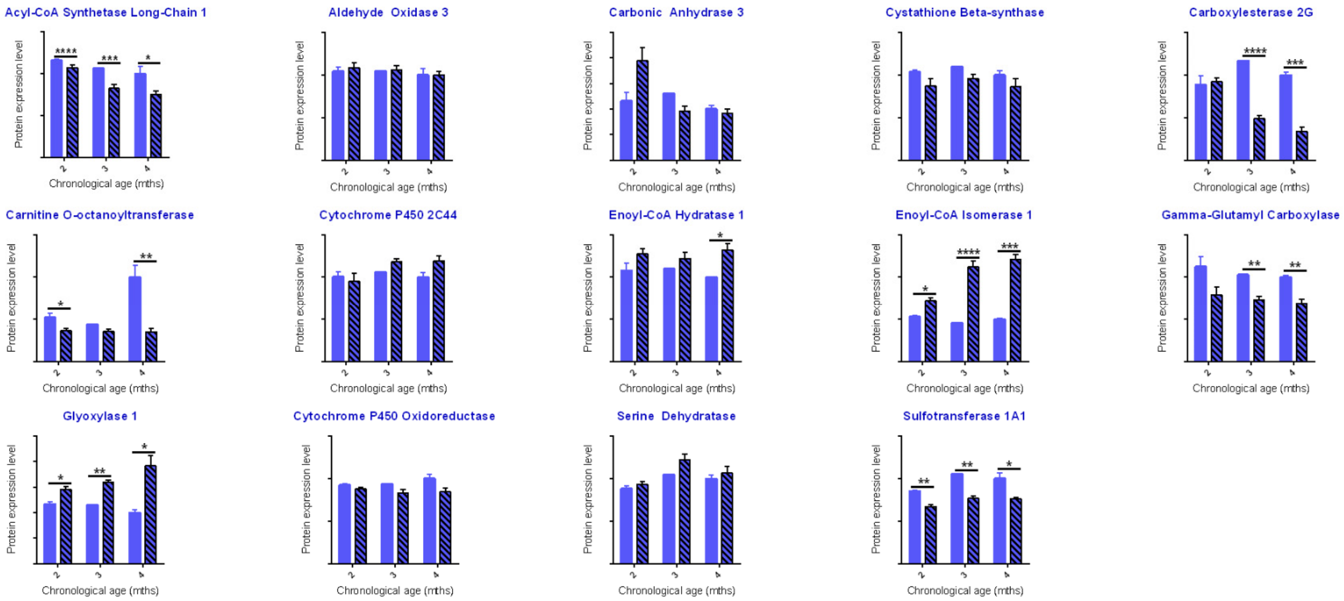

Supplementary Figure 8. Protein expression for select proteins in female f1b WT and female f1b *Ercc1*$^{-/\Delta}$ mouse liver. Expression of the proteins selected for the biological age calculator in female f1b C57BL/6J:FVB/NJ (blue) and female f1b C57BL/6J:FVB/NJ *Ercc1*$^{-/\square}$ (blue hatched) mouse liver at multiple ages. Error bars indicate SEM. *p<0.05, **p<0.01, ***p<0.001, ****p< 0.0001, one-way ANOVA.
