# Supplementary material for: Measuring biological age in mice using differential mass spectrometry
Source: Aging (Albany NY). 2019 Feb 11;11(3):1045–61. doi: 10.18632/aging.101810 (PMC6382423; doi:10.18632/aging.101810)
Supplement: Supplementary Tables [file aging-11-101810-s002.pdf]

Supplementary Table 1. Details on the number of liver samples used for the initial proteomics analysis.

**Genotype:**	**WT**	**WT**	**WT**	**WT**	**WT**	***Ercc1***$^{-/\square}$	***Ercc1***$^{-/\square}$
**Strain:**	C57B6/Jni<br>a<br>**inbred**	C57BL6/Jnia:<br>Balb/cBy<br>**f1 hybrid a**	C57BL/6J:<br>FVB/NJ<br>**f1 hybrid b**	C57BL/6J:<br>FVB/NJ<br>**f1 hybrid b**	C57BL/6J:<br>FVB/NJ<br>**f1 hybrid b**	C57BL/6J:<br>FVB/NJ<br>**f1 hybrid b**	C57BL/6J:<br>FVB/NJ<br>**f1 hybrid b**
**Sex:**	**Male**	**Male**	**Female**	**Male**	**Female**	**Male**	**Female**
**Age (mths):**							
**2**				3	2	3	5
**3**				1	2	8	5
**4**				3	3	5	7
**7**			9				
**8**	8	7					
**14**			6				
**16**	8	8					
**24**	7	8	7				
**30**			3				
**32**	8	8					
**36**		6					
**Total:**	31	37	25	7	7	16	17

Supplementary Table 2. The table delineates the number of liver samples used in the initial set of experiments. Quantified features are the number of mass spectrometric signals that passed occupancy and outlier filtering. Identified features are those with an identification associated with a peptide and a protein. One-way ANOVA analysis across the lifespan measures of the various age groups of samples is shown at varying significances. Minimum significance cutoff for a false discover rate of less than 5% is shown in red.

	**Total all groups**	**WT male inbred**	**WT male f1a**	**WT female f1b**	***Ercc1***$^{-/\square}$ **and WT male f1b**	***Ercc1***$^{-/\square}$ **and WT female f1b**
**Number of livers**	140	31	37	25	23	24
**Quantified features**		64,657	60,653	76,683	46,292	49,004
**Identified features**		34,817	34,301	35,434	28,647	30,290
**Quantified proteins**	1,489	1,298	1,268	1,196	1,181	1,238
**One-way ANOVA:**						
**p<0.05**		316	436	562	266	173
**p<0.01**		**127**	222	362	138	**69**
**p<0.001**		55	**92**	167	**46**	14
**p<0.0001**		23	44	**74**	19	3

Supplementary Table 3. All significant proteins for the male inbred C57BL/6Jnia as determined by one-way ANOVA analysis with a false discovery rate of 5%. Shown for each protein is the mean expression of that protein at a particular age relative to expression of that protein in C57BL/6Jnia male mice at 8 months of age. The mean expression level is not 1 for all proteins in the 8 month-old mice is that the expression levels were normalized at the feature level. Multiple features or peptides are combined to calculate the expression of a single protein. Therefore the protein expression indicated in the first column, which is the reference population, is not precisely 1.

	**8 months**		**16 months**		**24 months**		**32 months**	
	Mean	***SEM***	***Mean***	***SEM***	***Mean***	***SEM***	***Mean***	***SEM***
AASS	0.93	0.03	0.93	0.02	0.98	0.06	1.40	0.13
ACAA1B	1.15	0.07	1.17	0.09	0.95	0.12	0.78	0.06
ACAA2	1.06	0.02	1.03	0.02	0.94	0.04	0.88	0.06
ACAT1	1.06	0.04	1.07	0.03	0.97	0.02	0.87	0.05
ACO1	1.06	0.02	1.01	0.01	0.99	0.02	0.93	0.03
ACO2	0.95	0.02	0.98	0.02	0.98	0.03	1.11	0.03
ACP1	0.94	0.05	1.23	0.08	0.96	0.06	0.90	0.08
ACSL1	1.10	0.04	1.09	0.03	0.91	0.04	0.81	0.06
ACSM3	0.88	0.08	0.83	0.09	1.23	0.19	1.49	0.13
ACTB	0.91	0.03	0.97	0.02	1.16	0.11	1.32	0.13
ACTN1	0.92	0.01	0.94	0.02	1.15	0.10	1.22	0.08
ACTN4	0.87	0.04	0.92	0.03	1.12	0.10	1.26	0.10
ACTR1A	0.92	0.08	0.56	0.12	1.14	0.08	1.07	0.09
ACTR3	0.93	0.02	0.94	0.03	1.16	0.09	1.22	0.09
AGL	0.98	0.03	1.08	0.05	1.10	0.07	0.84	0.06
AKR1C13	1.02	0.04	1.06	0.03	1.05	0.06	0.82	0.04
AKR1C6	1.08	0.02	1.05	0.03	0.95	0.06	0.86	0.05
AMT	0.88	0.04	0.92	0.06	1.10	0.04	1.14	0.05
ANXA6	0.87	0.02	0.96	0.03	1.07	0.05	1.20	0.08
AOX3	1.28	0.06	1.10	0.07	0.89	0.03	0.66	0.07
ATP11C	1.06	0.06	1.03	0.03	0.97	0.05	0.79	0.06
ATP6V1B2	0.97	0.06	0.82	0.05	1.02	0.09	1.27	0.10
BC021614	1.42	0.07	0.95	0.09	0.86	0.13	0.69	0.07
C16ORF13	1.17	0.03	1.00	0.05	0.96	0.06	0.85	0.09
CA3	1.14	0.04	1.12	0.03	0.84	0.05	0.56	0.07
CBS	0.89	0.01	0.97	0.04	0.98	0.03	1.22	0.05
CCDC47	0.93	0.04	0.92	0.06	1.08	0.08	1.31	0.08
CERCAM	1.56	0.10	1.03	0.07	0.85	0.13	0.40	0.09
CES2G	1.10	0.04	1.04	0.02	0.98	0.05	0.79	0.05
CES3A	1.09	0.04	1.05	0.05	0.88	0.08	0.73	0.09
CFL1	0.94	0.02	0.97	0.03	1.12	0.06	1.13	0.05
COMT	1.14	0.03	1.00	0.04	0.92	0.05	0.90	0.07
CPA4	0.96	0.14	1.25	0.06	1.15	0.09	0.70	0.06
CROT	1.18	0.06	1.05	0.05	0.93	0.05	0.82	0.05
CTH	0.97	0.04	0.95	0.02	0.85	0.08	1.46	0.14

	8 months		16 months		24 months		32 months	
	**Mean**	**SEM**	**Mean**	**SEM**	**Mean**	**SEM**	**Mean**	**SEM**
CTSD	0.89	0.03	0.92	0.03	1.10	0.10	1.25	0.09
CYP2C44	0.92	0.03	0.87	0.04	1.07	0.07	1.27	0.06
CYP2C68	0.83	0.06	0.96	0.10	1.15	0.14	1.45	0.12
CYP2C70	0.85	0.06	0.86	0.08	1.05	0.07	1.41	0.12
CYP2D9	1.26	0.15	1.12	0.06	0.77	0.11	0.66	0.06
CYP2E1	1.01	0.02	1.10	0.05	0.96	0.05	0.85	0.06
CYP2F2	1.10	0.05	0.97	0.05	0.83	0.05	1.03	0.05
CYP4A12B	1.19	0.07	0.93	0.04	0.86	0.05	0.98	0.05
CYP4V2	1.27	0.11	1.15	0.13	0.67	0.08	0.84	0.08
DHCR7	0.81	0.06	0.97	0.07	1.17	0.07	1.20	0.10
DHTKD1	0.84	0.06	0.82	0.08	1.04	0.05	1.51	0.18
DPYD	0.85	0.04	0.99	0.03	1.08	0.08	1.31	0.11
ECH1	1.05	0.04	1.15	0.05	0.97	0.02	0.84	0.04
ECI1	1.07	0.03	1.09	0.03	0.88	0.05	0.80	0.05
EPHX2	1.15	0.03	1.09	0.04	0.89	0.06	0.71	0.05
FABP1	1.10	0.07	1.24	0.06	0.93	0.08	0.54	0.05
FDPS	0.75	0.06	1.05	0.10	1.34	0.11	1.00	0.06
FMO5	0.94	0.02	0.97	0.06	0.98	0.03	1.20	0.08
FN1	0.94	0.02	0.96	0.04	0.88	0.04	1.23	0.12
FTH1	0.92	0.09	0.84	0.10	1.23	0.19	1.81	0.20
FTL1	0.87	0.09	0.76	0.07	1.19	0.09	1.99	0.33
GADL1	1.26	0.12	1.23	0.18	1.46	0.20	0.67	0.05
GBE1	1.01	0.02	1.09	0.05	1.00	0.06	0.82	0.04
GGCX	0.83	0.04	0.96	0.04	1.23	0.13	1.17	0.07
GLDC	1.19	0.04	0.96	0.04	0.87	0.06	0.90	0.06
GLO1	1.08	0.04	1.12	0.03	0.98	0.05	0.72	0.05
GLT28D2	0.96	0.04	1.01	0.06	0.89	0.08	1.63	0.15
GM4952	1.14	0.09	1.17	0.07	0.97	0.08	0.76	0.05
GNPNAT1	0.90	0.03	1.00	0.05	1.01	0.04	1.13	0.04
GOT1	0.87	0.04	0.99	0.04	1.00	0.05	1.47	0.13
GPD1	1.08	0.03	1.11	0.05	0.98	0.05	0.85	0.03
GSTA1	0.94	0.02	1.10	0.04	1.03	0.05	0.92	0.03
GSTM1	0.92	0.05	0.92	0.03	1.03	0.05	1.20	0.09
GYK	0.96	0.02	1.13	0.04	1.01	0.04	0.96	0.03
GYS2	1.11	0.08	1.10	0.06	0.96	0.09	0.72	0.06
HMGCL	1.03	0.03	1.08	0.02	0.91	0.03	0.93	0.02
HMGCS2	1.06	0.04	1.09	0.03	0.89	0.04	0.92	0.06
HNRNPD	0.94	0.05	0.89	0.05	1.19	0.10	1.26	0.12
HSD17B2	1.14	0.06	1.03	0.07	0.96	0.04	0.77	0.07
INTS7	1.29	0.13	1.12	0.05	0.73	0.07	0.76	0.11
IQGAP2	0.91	0.02	0.99	0.02	1.00	0.03	1.15	0.05

IYD	0.95	0.04	0.91	0.05	1.05	0.07	1.30	0.09
	8 months		16 months		24 months		32 months	
	**Mean**	**SEM**	**Mean**	**SEM**	**Mean**	**SEM**	**Mean**	**SEM**
KEG1	1.15	0.04	0.99	0.06	0.92	0.06	0.84	0.08
KRT10	0.92	0.02	0.94	0.01	1.08	0.09	1.31	0.08
KRT8	0.91	0.02	0.96	0.02	1.10	0.06	1.26	0.07
LACTB2	1.10	0.04	1.03	0.03	0.97	0.03	0.88	0.05
LGALS3BP	0.84	0.10	0.88	0.08	0.95	0.06	1.65	0.22
LGALS9	0.87	0.05	0.90	0.06	1.14	0.03	1.20	0.09
MAN2A1	0.88	0.06	0.78	0.06	1.35	0.13	1.24	0.11
MAT1A	0.77	0.05	0.80	0.08	1.15	0.14	1.47	0.14
MAT2A	0.75	0.03	0.96	0.06	1.10	0.07	1.52	0.12
ME1	1.02	0.09	1.41	0.14	1.06	0.14	0.67	0.09
MUP10	1.27	0.08	1.24	0.10	0.78	0.06	0.57	0.07
MUP3	1.31	0.07	0.97	0.10	0.79	0.07	0.62	0.07
NNT	1.08	0.15	1.34	0.13	1.37	0.11	0.69	0.11
NPM1	0.90	0.03	1.05	0.04	1.37	0.18	0.87	0.05
NXN	0.70	0.06	1.05	0.09	1.23	0.13	1.54	0.27
OTC	1.07	0.03	0.98	0.03	0.90	0.04	0.98	0.03
PAICS	0.92	0.03	0.91	0.05	1.04	0.11	1.36	0.05
PEPD	0.99	0.05	0.90	0.08	0.95	0.10	1.30	0.09
PKLR	1.04	0.06	1.07	0.09	1.22	0.09	0.78	0.07
PLS3	0.88	0.02	0.99	0.03	1.03	0.04	1.15	0.06
PON1	0.95	0.05	0.93	0.05	0.95	0.06	1.26	0.06
POR	0.87	0.02	0.96	0.03	1.07	0.04	1.20	0.06
PPA1	0.99	0.02	0.94	0.02	1.00	0.04	1.12	0.04
PRDX3	0.94	0.02	0.97	0.04	0.98	0.04	1.09	0.03
PSMD1	0.93	0.06	0.84	0.05	1.17	0.09	1.06	0.07
PSME1	0.91	0.03	0.92	0.04	1.20	0.10	1.18	0.09
PTER	0.84	0.04	0.88	0.06	1.20	0.09	1.17	0.08
PYGB	1.08	0.04	1.06	0.04	1.01	0.06	0.80	0.05
RSU1	0.87	0.05	0.91	0.06	1.11	0.14	1.37	0.11
SCP2	1.05	0.03	1.06	0.03	0.89	0.06	0.80	0.08
SDS	0.93	0.05	0.87	0.03	1.02	0.06	1.44	0.11
SEPT7	2.27	0.45	0.73	0.12	1.14	0.20	1.10	0.12
SERPINA1A	1.25	0.11	1.06	0.06	0.93	0.08	0.76	0.06
SERPINA3G	1.20	0.12	1.12	0.11	0.92	0.10	0.62	0.08
SERPINH1	0.76	0.07	1.03	0.10	1.19	0.10	1.09	0.07
SLC27A5	0.92	0.02	0.96	0.02	1.00	0.04	1.13	0.05
SOD2	1.03	0.03	1.01	0.04	0.98	0.03	0.88	0.03
STEAP4	0.78	0.07	0.93	0.08	1.54	0.17	1.23	0.19
SULT1A1	0.70	0.03	0.97	0.03	1.14	0.07	1.46	0.09

SULT1D1	0.88	0.04	1.04	0.04	0.93	0.06	1.12	0.05
SULT2A3	1.10	0.05	1.14	0.06	0.86	0.07	0.68	0.08
TAGLN2	0.91	0.04	0.98	0.07	1.16	0.13	1.47	0.14
	**8 months**		**16 months**		**24 months**		**32 months**	
	**Mean**	**SEM**	**Mean**	**SEM**	**Mean**	**SEM**	**Mean**	**SEM**
TGM7	1.09	0.09	1.16	0.08	0.87	0.09	0.59	0.05
TMCO1	1.08	0.04	0.82	0.03	1.01	0.06	1.05	0.05
TSTD1	0.43	0.08	0.95	0.10	1.02	0.11	1.11	0.15
TWF1	0.91	0.04	0.90	0.05	1.08	0.09	1.26	0.11
UGT1A10	0.62	0.09	1.10	0.09	1.10	0.17	0.92	0.06
URAD	0.85	0.02	0.96	0.05	1.09	0.09	1.43	0.12
VDAC2	0.95	0.02	0.96	0.02	1.05	0.04	1.10	0.02
XYLB	0.92	0.02	0.96	0.02	1.06	0.03	1.05	0.02

Supplementary Table 4. All significant proteins for the male f1a C57BL6/Jnia:Balb/cBy via one-way ANOVA analysis with a false discovery rate of 5%. Shown for each protein is the mean expression of that protein at a particular age relative to expression of that protein in C57BL/6Jnia male mice at 8 months of age.

	**8 months**		**16 months**		**24 months**		**32 months**		**36 months**	
	Mean	SEM	Mean	SEM	Mean	SEM	Mean	SEM	Mean	SEM
9030617O03RIK	1.01	0.06	1.32	0.09	1.41	0.09	1.56	0.05	1.17	0.20
AARS	0.97	0.05	0.81	0.04	0.78	0.02	0.85	0.03	0.94	0.18
AASS	1.02	0.04	1.03	0.03	1.09	0.02	1.26	0.02	1.05	0.18
ACLY	0.99	0.08	0.94	0.05	0.90	0.05	0.74	0.03	1.15	0.22
ACSF2	1.05	0.02	1.02	0.03	1.12	0.02	1.09	0.02	0.77	0.11
ACSM1	1.05	0.05	1.04	0.03	1.07	0.02	1.24	0.01	0.85	0.13
ACTN1	1.00	0.01	1.07	0.02	1.13	0.02	1.29	0.02	1.23	0.23
ALDH1A7	1.02	0.04	1.01	0.02	1.02	0.03	1.05	0.03	0.69	0.12
ALDH3A2	1.04	0.03	1.09	0.03	1.05	0.04	1.00	0.01	0.64	0.11
ALDH7A1	1.04	0.03	1.04	0.02	1.15	0.03	1.11	0.02	0.83	0.13
ANXA6	1.04	0.02	1.08	0.02	1.10	0.02	1.15	0.03	1.18	0.19
AP2M1	1.01	0.07	1.11	0.04	0.87	0.06	1.20	0.03	1.31	0.24
ARG1	0.98	0.03	1.02	0.03	1.02	0.04	1.05	0.01	1.07	0.20
AS3MT	0.98	0.09	0.84	0.09	1.06	0.07	1.63	0.16	2.21	0.51
ASAH1	0.97	0.09	0.87	0.08	1.21	0.09	1.56	0.05	1.15	0.23
ASL	1.03	0.01	1.04	0.02	0.97	0.04	0.97	0.02	1.06	0.18
ATP1B1	0.98	0.05	1.07	0.03	1.12	0.06	1.20	0.04	0.70	0.14
ATP2A2	1.04	0.09	1.22	0.08	0.95	0.11	1.64	0.10	1.73	0.36
CA2	0.98	0.04	1.23	0.07	1.35	0.10	0.96	0.03	0.81	0.15
CA3	1.02	0.03	1.02	0.05	1.00	0.04	0.79	0.02	0.54	0.11
CBS	1.01	0.05	1.08	0.04	1.03	0.03	1.25	0.04	1.17	0.20
CCDC66	0.99	0.09	1.53	0.17	1.64	0.11	0.84	0.07	0.78	0.15
CYP2A12	1.03	0.14	0.82	0.06	0.73	0.06	1.56	0.03	1.38	0.33

CYP2C29	0.99	0.07	1.18	0.10	0.99	0.12	1.07	0.09	0.31	0.08
CYP2C40	0.97	0.04	1.02	0.03	0.97	0.07	1.16	0.05	0.49	0.10
CYP2C44	0.99	0.05	1.07	0.07	1.15	0.06	1.57	0.06	1.17	0.23
CYP2C69	0.96	0.06	1.05	0.04	0.99	0.05	1.10	0.03	0.55	0.12
CYP3A11	0.97	0.05	0.78	0.02	0.78	0.06	0.83	0.04	0.42	0.09
CYP4A12B	1.03	0.03	0.99	0.05	1.12	0.04	1.08	0.01	0.72	0.12
CYP7B1	1.02	0.13	1.09	0.07	1.81	0.18	1.72	0.09	0.71	0.19
DDX1	0.95	0.08	1.13	0.10	1.14	0.11	1.22	0.06	1.89	0.36
DECR1	0.98	0.03	0.98	0.03	0.96	0.05	0.92	0.01	0.58	0.11
DPYD	0.96	0.06	1.11	0.05	1.14	0.06	1.25	0.05	1.18	0.22
ECHS1	1.01	0.03	1.02	0.02	1.07	0.02	1.17	0.02	0.93	0.15
ELOVL5	0.97	0.13	0.96	0.05	0.92	0.06	0.74	0.03	1.23	0.35
EPHX2	1.04	0.03	0.97	0.03	0.91	0.03	0.80	0.03	0.73	0.12
FTH1	1.03	0.04	0.82	0.09	0.69	0.10	1.32	0.13	2.66	0.59
FTL1	0.98	0.07	0.99	0.07	1.08	0.08	1.52	0.11	2.47	0.56
	8 months		16 months		24 months		32 months		36 months	
	Mean	SEM	Mean	SEM	Mean	SEM	Mean	SEM	Mean	SEM
GADL1	0.98	0.10	0.78	0.09	0.88	0.06	0.96	0.07	1.65	0.29
GCDH	1.05	0.02	1.07	0.03	1.03	0.02	0.99	0.01	0.74	0.11
GCK	1.02	0.04	0.88	0.04	0.96	0.04	0.91	0.04	1.13	0.22
GGCX	1.02	0.10	1.03	0.05	1.10	0.05	1.35	0.04	1.74	0.32
GOT1	0.97	0.05	1.11	0.08	1.01	0.06	1.10	0.06	1.24	0.22
GULO	1.01	0.01	0.95	0.03	0.86	0.03	0.97	0.03	0.65	0.11
HAL	0.98	0.04	0.93	0.02	0.88	0.03	0.85	0.03	0.94	0.17
HBA	1.04	0.08	1.33	0.13	1.43	0.11	0.93	0.03	0.76	0.13
HBBT1	1.04	0.06	1.47	0.15	1.58	0.12	0.96	0.04	0.78	0.13
HNRNPA2B1	1.05	0.03	1.06	0.03	1.11	0.01	1.19	0.03	1.17	0.20
HNRNPA3	0.97	0.01	0.96	0.02	1.03	0.04	1.11	0.03	1.06	0.20
HSD17B13	1.03	0.04	1.03	0.05	1.10	0.07	1.06	0.04	1.45	0.30
KEG1	0.99	0.02	0.92	0.03	1.07	0.03	0.98	0.02	0.69	0.12
KRT10	0.98	0.04	1.09	0.03	1.15	0.03	1.45	0.05	1.42	0.29
KRT8	1.04	0.03	1.10	0.03	1.25	0.04	1.41	0.05	1.36	0.24
LDHA	1.00	0.03	0.97	0.03	0.90	0.02	1.00	0.02	1.08	0.20
LGALS3BP	0.96	0.10	0.95	0.08	0.89	0.06	1.75	0.03	0.84	0.26
LPCAT3	1.02	0.05	1.13	0.04	1.14	0.05	1.15	0.01	1.18	0.23
MARC2	0.96	0.04	1.00	0.04	1.01	0.04	1.09	0.03	0.62	0.12
MATR3	1.04	0.05	1.00	0.07	1.05	0.07	1.25	0.04	1.49	0.29
ME1	1.02	0.11	0.99	0.08	0.87	0.08	0.45	0.03	0.86	0.17
MUP10	1.00	0.07	1.02	0.07	0.97	0.05	0.79	0.03	0.42	0.09
MYO18A	0.99	0.15	1.11	0.08	1.05	0.08	1.80	0.08	2.23	0.57
NAMPT	1.00	0.07	0.79	0.04	1.24	0.05	1.37	0.05	1.65	0.40
NAPRT1	1.02	0.05	1.07	0.02	1.00	0.04	1.09	0.02	1.10	0.18
PAICS	0.97	0.10	0.98	0.05	1.17	0.07	1.40	0.05	1.36	0.25
PAQR9	1.02	0.08	1.03	0.08	1.01	0.12	1.25	0.09	1.42	0.30

PCYT2	1.05	0.05	1.05	0.08	1.07	0.04	1.00	0.02	1.25	0.20
PECR	1.02	0.02	1.06	0.03	1.02	0.02	0.99	0.01	0.71	0.12
PGLS	1.02	0.04	1.04	0.03	1.03	0.05	0.94	0.04	1.12	0.19
PLS3	0.99	0.03	1.04	0.02	1.12	0.04	1.26	0.07	1.22	0.23
PON1	0.96	0.05	0.97	0.04	0.95	0.03	1.21	0.04	0.54	0.11
PRDX4	1.01	0.02	0.99	0.02	1.02	0.02	1.10	0.03	0.99	0.16
QPRT	0.98	0.03	0.92	0.02	0.90	0.03	0.96	0.02	1.06	0.19
RAB1A	0.97	0.04	1.00	0.04	0.87	0.05	1.17	0.04	1.08	0.20
RPL35A	1.01	0.07	1.04	0.06	1.01	0.06	1.16	0.07	1.37	0.27
RPS26	0.96	0.04	0.83	0.02	0.83	0.02	0.87	0.04	1.01	0.20
RPS4X	0.97	0.04	0.94	0.02	0.90	0.01	1.03	0.02	1.01	0.19
SARDH	0.98	0.03	0.99	0.02	1.00	0.03	0.96	0.02	0.59	0.11
SCARA3	0.99	0.05	1.11	0.06	1.08	0.08	0.94	0.07	0.53	0.10
SCD1	1.00	0.16	0.98	0.17	0.88	0.09	0.54	0.06	2.28	0.70
SDS	0.98	0.04	1.14	0.05	1.06	0.04	1.18	0.05	1.61	0.30
	8 months		16 months		24 months		32 months		36 months	
	Mean	SEM	Mean	SEM	Mean	SEM	Mean	SEM	Mean	SEM
SEC14L4	1.03	0.04	1.10	0.02	1.11	0.02	0.98	0.02	1.15	0.19
SELENBP2	1.04	0.10	1.04	0.09	1.37	0.08	1.57	0.02	1.16	0.20
SERPINA3G	0.97	0.07	1.15	0.10	1.35	0.12	0.79	0.04	0.70	0.13
SLC4A1	1.00	0.04	1.41	0.17	1.63	0.14	0.91	0.05	0.76	0.13
SORD	0.99	0.02	0.97	0.02	0.90	0.03	0.93	0.02	0.69	0.12
SPR	1.00	0.05	0.99	0.03	1.03	0.03	1.11	0.03	1.06	0.18
SULT2A3	1.02	0.04	1.08	0.05	0.98	0.04	0.96	0.03	0.63	0.11
TKT	0.99	0.02	1.00	0.03	1.03	0.01	1.00	0.02	1.05	0.18
TMEM205	1.01	0.04	1.00	0.03	1.09	0.05	1.11	0.04	0.72	0.12
TPI1	1.01	0.02	0.97	0.02	1.00	0.02	1.07	0.01	1.07	0.18
UGT1A5	0.98	0.02	0.98	0.01	1.01	0.03	1.03	0.01	0.76	0.13
UGT3A2	0.97	0.03	0.93	0.02	0.93	0.03	0.98	0.04	0.58	0.11

Supplementary Table 5. All significant proteins for the female f1b C57BL/6J:FVB/NJ via one-way ANOVA analysis with a false discovery rate of 5%. Shown for each protein is the mean expression of that protein at a particular age relative to expression of that protein in C57BL/6Jnia male mice at 8 months of age.

	**8 months**		**14 months**		**24 months**		**30 months**	
	Mean	SEM	Mean	SEM	Mean	SEM	Mean	SEM
ABCC6	1.09	0.05	1.18	0.07	0.89	0.07	0.73	0.03
ACAA1B	0.78	0.05	1.29	0.11	1.53	0.09	0.70	0.11
ACAT1	0.97	0.05	1.19	0.07	1.08	0.04	0.59	0.11
ACOX1	0.88	0.04	1.19	0.05	1.12	0.05	0.79	0.08
ACOX2	0.86	0.05	1.03	0.05	1.07	0.08	1.94	0.33
ACTN1	0.99	0.03	1.03	0.06	0.93	0.04	1.53	0.14
ADCK3	1.02	0.06	1.18	0.08	0.92	0.06	0.40	0.06
ADH4	0.97	0.06	1.10	0.12	0.93	0.08	1.69	0.15
AGXT2	0.95	0.03	1.08	0.05	1.02	0.02	0.79	0.03
AHNAK	0.96	0.07	1.05	0.09	0.85	0.07	1.54	0.08
AK2	1.04	0.05	1.14	0.06	0.97	0.03	0.68	0.09
ALDH9A1	1.00	0.03	1.13	0.07	1.07	0.02	0.64	0.02
ANXA11	1.61	0.37	0.91	0.30	0.80	0.33	6.22	0.23
APOB	1.00	0.06	0.96	0.08	1.01	0.07	1.63	0.05
APRT	1.05	0.05	1.09	0.14	0.95	0.02	3.60	0.16
ARCN1	1.00	0.07	1.02	0.06	0.93	0.07	1.82	0.08
AS3MT	0.96	0.13	0.94	0.14	1.09	0.12	2.36	0.26
BDH1	0.88	0.04	1.12	0.03	1.08	0.03	0.60	0.14
BPHL	0.95	0.04	1.11	0.05	1.08	0.05	0.73	0.06
CA3	1.02	0.04	1.15	0.06	1.03	0.03	0.60	0.05
CES2G	0.97	0.02	1.20	0.07	0.97	0.04	0.64	0.15
CP	0.93	0.07	0.88	0.09	0.99	0.08	4.31	0.97
CRYZ	0.90	0.02	1.11	0.05	1.17	0.05	0.87	0.05
DDX39A	0.97	0.01	1.06	0.06	0.94	0.05	1.68	0.23
FBP1	0.96	0.03	1.10	0.06	1.11	0.03	0.74	0.09
FDX1	1.42	0.08	0.91	0.06	0.93	0.04	0.76	0.03
FGGY	0.80	0.08	1.19	0.14	0.95	0.10	1.65	0.13
FMO1	1.22	0.06	1.08	0.07	0.82	0.04	0.55	0.09
GALK1	0.87	0.08	0.94	0.06	1.12	0.05	1.45	0.05
GC	0.92	0.07	1.02	0.09	1.04	0.04	1.59	0.15
GCLC	0.94	0.03	1.19	0.07	1.10	0.07	0.65	0.09
GNB2	0.97	0.06	0.98	0.05	0.95	0.01	1.41	0.08
GSS	1.06	0.04	0.99	0.07	0.91	0.06	1.84	0.27
GSTZ1	0.98	0.04	1.11	0.06	1.06	0.03	0.78	0.02
HDHD2	1.01	0.04	1.17	0.08	1.03	0.04	0.65	0.08
HNRNPA2B1	0.93	0.04	0.99	0.05	1.03	0.05	1.31	0.02
HNRNPAB	1.00	0.04	1.01	0.07	1.05	0.05	1.72	0.19
HNRNPD	0.93	0.03	0.99	0.04	0.95	0.06	1.96	0.30
	8 months		14 months		24 months		30 months	

	Mean	SEM	Mean	SEM	Mean	SEM	Mean	SEM
HNRNPK	0.99	0.02	0.99	0.04	0.98	0.02	1.39	0.14
IQGAP2	0.90	0.05	1.09	0.09	0.96	0.04	1.49	0.09
MAOB	1.08	0.03	1.07	0.08	0.98	0.04	0.68	0.06
MTHFD1	0.98	0.03	1.14	0.05	1.03	0.03	0.73	0.05
MYO1C	0.91	0.05	1.04	0.07	1.01	0.07	1.70	0.24
NCL	0.98	0.04	0.97	0.05	0.95	0.09	2.05	0.34
NQO2	0.91	0.07	1.09	0.10	1.34	0.08	0.50	0.16
NUDT7	1.38	0.08	1.07	0.11	0.90	0.03	0.44	0.04
PDCD6	0.98	0.08	0.90	0.11	0.87	0.05	1.48	0.06
PKLR	0.86	0.05	1.12	0.09	1.59	0.18	0.55	0.15
PLEC	1.07	0.07	0.89	0.05	0.83	0.04	4.06	0.54
PPP6C	1.03	0.05	0.89	0.10	0.98	0.05	1.45	0.06
PRPS1L3	0.84	0.07	1.25	0.08	1.15	0.03	0.64	0.10
PSMC2	0.97	0.06	1.06	0.09	0.99	0.06	1.95	0.27
PSMC6	0.94	0.03	1.11	0.04	0.99	0.08	1.47	0.09
RGN	1.04	0.04	1.13	0.05	0.99	0.04	0.69	0.09
RPL12	1.00	0.03	0.99	0.09	0.98	0.03	1.47	0.10
RPL24	0.93	0.06	1.05	0.10	0.95	0.03	1.66	0.21
RPL27A	0.85	0.04	0.98	0.04	0.92	0.05	1.31	0.03
RPS10	0.99	0.03	1.06	0.09	0.99	0.03	1.49	0.12
RPS6	1.00	0.04	1.01	0.06	0.92	0.05	1.62	0.23
RPS8	1.02	0.01	1.04	0.08	0.95	0.02	1.51	0.15
SARDH	1.08	0.05	1.09	0.06	0.98	0.06	0.65	0.07
SEC14L2	0.99	0.03	1.13	0.03	0.99	0.04	0.55	0.09
SEC63	0.88	0.04	1.01	0.10	0.97	0.06	1.98	0.35
SLC22A18	0.84	0.05	1.19	0.13	0.96	0.07	1.71	0.15
SND1	1.00	0.03	1.03	0.09	0.96	0.02	1.49	0.15
SNX2	0.83	0.04	1.13	0.04	1.13	0.06	0.76	0.09
SOD1	0.96	0.06	1.20	0.09	1.18	0.09	0.58	0.09
SORD	1.02	0.02	1.11	0.04	0.99	0.03	0.73	0.09
STARD5	1.06	0.10	0.98	0.11	0.94	0.06	1.77	0.15
TPP1	0.99	0.05	0.96	0.09	0.92	0.06	2.11	0.43
TTC36	1.09	0.04	1.05	0.03	0.90	0.07	0.65	0.10
TWF1	1.01	0.04	1.03	0.09	0.93	0.03	2.06	0.41
TXNDC5	0.99	0.06	1.01	0.04	0.94	0.08	1.78	0.12
VTN	0.98	0.04	1.08	0.17	0.93	0.10	2.32	0.38

Supplementary Table 6. Pathways identified as altered most significantly with chronological age. Tables show $\log_2$ fold difference in protein expression of all proteins significantly altered with chronological age found in pathways identified as being altered with aging in three wild-type strains of mice. The numbers indicate the ratio of old vs. young for male **inbred** C57BL/6Jnia (M1), male **f1a** C57BL6/Jnia:Balb/cBy (M2), and female **f1b** C57BL/6J:FVB/NJ (F) mice. Gold is used to highlight proteins where protein expression was greater in old mice than young. Blue is used to indicate proteins where expression was decreased in old mice compared to young. The darker the shade, the more expression differed between young and old mice. Bold is used to indicate when *p* is <0.05; bold and italics *p*<0.01; bold, italics and underline *p*<0.001 by one-way ANOVA.

H2O2 Antioxidant Response

	M1	M2	F
CAT	-0.15	-0.04	**-0.32**
FTH1	***0.99***	***1.61***	***1.19***
FTL1	***1.19***	***1.54***	***0.93***
GPX1	-0.19	-0.12	***-0.57***
SOD1	-0.23	0.08	***-0.74***
SOD2	***-0.23***	-0.10	0.04

Oxidative Protein Damage Response

	M1	M2	F
CBR1	**0.50**	**0.54**	0.27
PRDX3	***0.22***	0.20	0.17
TXN2	0.18	0.00	***-0.61***
TXNRD2	0.24	0.29	***0.33***

Fatty acid Oxidation

	M1	M2	F
ABCD3	-0.27	0.04	-0.12
ACAA1B	***-0.57***	-0.20	-0.15
ACADL	-0.03	-0.02	0.05
ACADS	-0.17	**-0.17**	0.00
ACADSB	***-0.46***	***0.57***	-0.82
ACAT1	**-0.28**	-0.13	***-0.70***
ACOX1	**-0.33**	-0.10	-0.16
ACSL1	***-0.43***	-0.21	***-0.42***
CPT2	-0.16	-0.10	0.05
ECH1	***-0.32***	0.32	-0.05
ECHS1	0.10	0.11	0.03
EHHADH	-0.07	-0.02	-0.15
GCDH	**-0.20**	***-0.31***	**-0.30**
HADHA	-0.13	-0.08	-0.13
HADHB	-0.16	-0.04	-0.09

Glutathione Production

	M1	M2	F
AHCY	-0.05	**-0.12**	0.14
CBS	***0.46***	***0.43***	**0.53**
CTH	***0.59***	0.03	-0.01
GCLC	**-0.29**	-0.10	***-0.52***
GSR	0.39	0.37	**0.50**
GSS	**0.42**	**0.74**	***0.80***
GSTM1	**0.39**	0.32	0.43
H6PD	0.20	0.22	***1.16***
MAT1A	***1.03***	**0.70**	***0.95***
PGD	0.21	0.57	0.29

FXR-RXR

	M1	M2	F
FASN	0.10	0.41	***-1.38***
PKLR	**-0.41**	0.03	**-0.63**
SERPINA1A	***-0.71***	***-0.42***	0.09
SULT2A3	***-0.70***	***-0.49***	0.29

CAR-RxR

	M1	M2	F
ALDH3A2	-0.17	***-0.53***	-0.05
ALDH4A1	0.06	-0.11	**-0.38**
ALDH5A1	***-0.39***	**-0.34**	-0.06
ALDH9A1	-0.10	-0.11	***-0.64***
CYP3A11	**-0.42**	***-0.92***	0.20
FMO1	0.00	***-0.37***	***-1.16***

PXR-RXR

	M1	M2	F
CYP1A2	***-0.40***	-0.29	-0.17
MAOB	0.14	-0.01	***-0.66***
SULT1A1	***1.06***	**0.58**	**0.36**

NFKB

	M1	M2	F
C3	***0.33***	0.77	**0.82**
FN1	**0.39**	0.35	**0.78**
FTH1	***0.99***	***1.61***	***1.19***
SOD2	***-0.23***	-0.10	0.04

CEBP

	M1	M2	F
APCS	0.38		**3.04**
CP	0.33	1.25	***2.21***
FTL1	***1.19***	***1.54***	***0.93***
HP	***0.63***	0.11	
HPX	0.54	0.78	
SERPINA1A	***-0.71***	***-0.42***	0.09

STAT3

	M1	M2	F
FGA	-0.32	0.32	***1.62***
FGB	-0.33	0.26	**0.81**
FGG	-0.27	0.37	0.03
HMOX1	-0.29	-0.20	***1.06***

FcγR Mediated Phagocytosis

	M1	M2	F
CDC42	0.37	0.21	0.48
MYO1B	0.09	0.23	**0.68**
MYO1C	0.52	0.66	***0.90***
TLN1	**0.31**	**0.46**	***0.74***
VAPB	0.12	0.08	***0.43***

PPAR-RXR

	M1	M2	F
ACOX1	**-0.33**	-0.10	-0.16
FABP1	***-1.03***	**-0.34**	**-0.99**
PEX11A	-0.67	0.54	-0.49

Clathrin Mediated Endocytosis

	M1	M2	F
ACTB	***0.53***	**0.58**	**0.89**
ACTN1	***0.40***	***0.51***	***0.63***
ACTN4	***0.53***	**0.44**	
ACTR2	**0.40**	0.32	**0.53**
ACTR3	***0.39***	0.62	**0.98**
AP2a2	**0.25**	**0.38**	0.28
AP2B1	0.41	0.18	**0.67**
AP2m1	0.15	***0.60***	**0.44**
ARF6	0.20	0.21	**0.42**
ARPC1A	0.16	0.46	**1.43**
ARPC1B	0.43	***1.09***	
ARPC2	0.38	**0.48**	**0.71**
ARPC3	0.00	0.15	**0.52**
ARPC4	0.27	0.67	***0.86***
ARPC5	0.41	**0.46**	
CLTA	0.27	-0.01	**0.79**
CLTB	**0.38**	0.75	**0.44**
CLTC	**0.36**	**0.39**	***0.65***
CTTN	**0.48**	***0.55***	
DNM3	0.27	0.51	**0.67**
ITGB1	***0.40***	**0.32**	0.04
PICALM	0.04	0.33	**0.50**
RAB11A	0.36	0.28	**0.57**
RAB5C	0.31	0.24	**0.59**
RAB7A	0.12	0.13	0.08
TF	0.42	0.47	**1.10**

PPAR

	M1	M2	F
CYP2C44	***0.47***	**0.46**	0.32
CYP2C70	***0.73***	***0.59***	0.30

Supplementary Table 7. VO_2 (ml/gm lean muscle) at rest or while active. Shown is the mean for 16-22 mice per strain and age.

Strain		4 months	12 months	20 months	28 months
C57BL/6Jnia (inbred)	resting	4301	4363	4290	3943
C57BL/6Jnia:Balb/cBy (f1a)	resting	4120	4058	4033	4188
C57BL/6Jnia (inbred)	active	4737	4845	4672	4380
C57BL/6Jnia:Balb/cBy (f1a)	active	4637	4496	4362	4465

Supplementary Table 8. Total composite lesion score (CLS) for inbred C57BL/6Jnia male mice and f1a hybrid C57BL/6Jnia:Balb/cBy mice at four ages. The total CLS represents the sum of the average histopathologic score from 4 organs (heart, lung, kidney, liver) of n=8 mice per strain and age group. Shown is the mean ± standard deviation, unpaired two-tailed Student's t test.

Strain	8 months	16 months	24 months	32 months
C57BL/6Jnia	5.3 ± 1.3	4.8 ± 1.7	15.7 ±2.1	20.8 ±1.0
C57BL/6Jnia:Balb/cBy	4.0 ±1.0	5.9 ±1.4	11.5 ±1.0	17.8 ±2.4
p value	0.14	0.40	0.011	0.036

Supplementary Table 9. Composite lesion score (CLS) on four tissues from inbred C57BL/6Jnia male mice and f1a hybrid C57BL/6Jnia:Balb/cBy mice at four ages. Shown is the mean ± standard deviation (n=8 mice per strain), unpaired two-tailed Student's t test. ns not significant.

Strain	8 months	16 months	24 months	32 months
**HEART**				
C57BL/6Jnia	0.9 ± 0.5	1.0 ± 0.75	4.0 ± 1.1	4.9 ± 1.2
C57BL/6Jnia:Balb/cBy	0.6 ± 0.7	1.3 ± 1.0	3.3 ± 0.4	4.4 ± 1.2
p value	ns	ns	0.090	ns
**LUNG**				
C57BL/6Jnia	1.6 ± 0.5	1.5 ± 0.9	3.1 ± 0.8	4.5 ± 0.9
C57BL/6Jnia:Balb/cBy	0.8 ± 0.7	1.4 ± 0.7	2.8 ± 0.4	5.0 ± 0.5
p value	0.014	ns	ns	ns
**LIVER**				
C57BL/6Jnia	1.5 ± 0.8	1.1 ± 0.4	5.0 ± 1.4	5.6 ± 0.6
C57BL/6Jnia:Balb/cBy	1.3 ± 0.4	1.3 ± 0.5	3.1 ± 0.5	4.2 ± 0.9
p value	ns	ns	0.0022	0.0007
**KIDNEY**				
C57BL/6Jnia	1.3 ± 0.7	1.3 ± 1.0	3.5 ± 0.9	5.5 ± 1.1
C57BL/6Jnia:Balb/cBy	1.1 ± 0.5	1.3 ± 1.1	2.1± 0.8	3.8 ± 1.2
p value	ns	ns	0.0075	0.0064

Supplementary Table 10. All significant proteins for the male *Ercc1*$^{-/}$[illegible] f1b C57BL/6J:FVB/NJ via one-way ANOVA analysis with a false discovery rate of 5%. Shown for each protein is the mean expression of that protein at a particular age relative to expression of that protein in C57BL/6Jnia male mice at 8 months of age.

	**2 months**		**3 months**		**4 months**	
	Mean	***SEM***	***Mean***	***SEM***	***Mean***	***SEM***
AGXT2	0.97	0.02	1.11	0.02	1.18	0.03
AOX3	1.22	0.05	0.95	0.04	0.92	0.05
ATP5I	1.19	0.07	0.96	0.04	0.86	0.03
CA3	1.19	0.06	0.82	0.03	0.75	0.02
CAT	1.16	0.04	1.02	0.03	0.95	0.03
CCT2	1.19	0.09	1.11	0.02	0.90	0.06
CERCAM	1.19	0.12	0.50	0.06	0.64	0.10
CERS2	1.31	0.05	0.97	0.08	0.90	0.05
CES3A	1.27	0.01	1.11	0.04	0.95	0.04
COMT	1.34	0.04	1.01	0.04	1.01	0.04
COX7A2	1.18	0.03	0.96	0.03	0.96	0.02
CTNNA1	1.45	0.12	0.87	0.05	0.91	0.09
EEF2	1.15	0.02	0.97	0.02	0.98	0.01
EGFR	1.08	0.03	0.91	0.04	0.81	0.02
ELAVL1	1.31	0.05	0.81	0.06	1.02	0.04
FABP1	1.39	0.17	0.82	0.06	0.79	0.06
FBP1	1.21	0.03	0.96	0.03	0.89	0.04
GBE1	1.08	0.04	0.77	0.04	0.88	0.03
GLUL	1.16	0.04	0.78	0.03	0.94	0.01
GM5745	1.17	0.04	0.99	0.02	1.01	0.03
GPD2	1.56	0.07	0.97	0.04	1.00	0.04
H3F3A	1.31	0.07	0.87	0.06	1.00	0.05
HIST1H1E	1.37	0.09	0.83	0.04	0.95	0.06
HIST1H2AL	1.31	0.03	0.81	0.06	0.83	0.09
HIST1H4A	1.33	0.06	0.81	0.03	0.93	0.05
HSP90B1	1.17	0.03	0.98	0.02	0.91	0.04
HSPA1L	1.14	0.01	1.01	0.02	1.01	0.02
HYOU1	1.18	0.02	1.00	0.01	0.93	0.03
INMT	1.36	0.17	0.77	0.05	0.58	0.05
IVD	0.85	0.06	1.13	0.03	1.21	0.05
LACTB2	1.06	0.02	0.89	0.01	0.84	0.02
LDHD	0.79	0.01	1.15	0.05	1.07	0.04
MSRA	1.05	0.04	0.82	0.03	0.76	0.05
MUP10	1.35	0.09	0.92	0.03	0.67	0.07
NUDT7	1.37	0.06	0.98	0.04	0.86	0.04
PAQR9	1.60	0.04	0.81	0.08	0.98	0.06

PCYT2	1.24	0.07	0.83	0.06	0.97	0.06
PSMB4	1.22	0.01	1.00	0.05	0.96	0.05
	**2 months**		**3 months**		**4 months**	
	Mean	***SEM***	***Mean***	***SEM***	***Mean***	***SEM***
PYGB	1.08	0.03	0.88	0.03	0.87	0.03
RPL13A	1.21	0.06	0.90	0.04	1.00	0.04
RPN2	1.16	0.04	1.00	0.02	0.90	0.03
RPS20	1.21	0.04	0.91	0.03	0.95	0.06
TF	1.15	0.04	0.86	0.05	0.89	0.04
THRSP	1.76	0.23	0.73	0.06	0.78	0.09
UGDH	0.87	0.06	1.20	0.05	1.23	0.05
UOX	1.08	0.04	0.86	0.06	0.75	0.04

Supplementary Table 11. All significant proteins for the female *Ercc1*$^{-/}$[illegible] f1b C57BL/6J:FVB/NJ via one-way ANOVA analysis with a false discovery rate of 5%. Shown for each protein is the mean expression of that protein at a particular age relative to expression of that protein in C57BL/6Jnia male mice at 8 months of age.

	**2 months**		**3 months**		4 months	
	Mean	***SEM***	***Mean***	***SEM***	***Mean***	***SEM***
9030617O03RIK	0.93	0.05	1.28	0.05	1.09	0.03
AACS	1.26	0.07	0.92	0.16	0.79	0.04
AASS	0.73	0.08	1.17	0.11	1.41	0.16
ABCC3	0.85	0.09	1.45	0.13	1.24	0.12
ACAD8	0.86	0.06	1.10	0.05	1.13	0.05
ACAT1	1.08	0.07	0.97	0.05	0.65	0.08
ACOX2	0.89	0.05	1.38	0.11	1.49	0.08
ACSL1	1.07	0.03	0.87	0.03	0.72	0.04
AKR1D1	0.94	0.04	1.16	0.05	1.08	0.03
ALDH3A2	0.71	0.09	1.22	0.09	1.16	0.11
ALDH7A1	0.96	0.03	1.07	0.02	1.16	0.03
ALDH9A1	0.87	0.02	0.99	0.04	0.99	0.01
ASPDH	0.97	0.04	0.94	0.03	0.74	0.03
ATP6V1A	0.99	0.03	1.03	0.05	1.25	0.07
BLMH	1.05	0.04	0.94	0.03	0.54	0.10
CA3	1.42	0.07	0.79	0.04	0.52	0.08
CAT	1.09	0.05	1.00	0.03	0.83	0.03
CBR1	0.78	0.03	1.17	0.12	1.30	0.11
CCBL1	1.11	0.06	0.90	0.08	0.71	0.05
CDH2	0.79	0.06	0.81	0.07	1.70	0.25
CES3A	1.74	0.23	0.91	0.09	0.82	0.07

CTH	0.88	0.05	1.07	0.06	1.41	0.14
CTSD	0.79	0.07	0.94	0.06	1.16	0.05
CYB5A	0.92	0.03	1.21	0.09	1.14	0.03
CYP2J5	0.94	0.03	1.12	0.01	1.17	0.06
CYP3A13	0.87	0.05	1.23	0.08	1.13	0.04
DHRS1	0.86	0.04	1.20	0.07	1.19	0.08
DLAT	0.76	0.06	1.04	0.04	1.05	0.07
DMGDH	0.84	0.03	0.97	0.04	1.04	0.03
DPYD	1.07	0.05	0.78	0.08	0.79	0.06
ECHDC2	0.92	0.04	1.14	0.03	1.31	0.09
ENTPD5	0.85	0.05	1.32	0.10	1.45	0.16
EPHX1	0.91	0.04	1.36	0.10	1.34	0.12
FMO5	0.92	0.05	1.43	0.05	2.00	0.23
GLUL	1.10	0.05	0.78	0.07	0.81	0.04
GPT2	0.97	0.03	1.12	0.01	1.25	0.07
GSTM1	0.92	0.04	1.30	0.09	1.47	0.16
GSTT3	0.79	0.03	0.89	0.07	1.12	0.03
	**2 months**		**3 months**		**4 months**	
	Mean	***SEM***	***Mean***	***SEM***	***Mean***	***SEM***
GYK	0.93	0.03	1.18	0.05	1.24	0.05
HIBADH	0.74	0.08	1.07	0.03	1.05	0.07
HNMT	0.89	0.05	1.01	0.05	1.34	0.03
HSD17B13	0.95	0.01	1.18	0.04	1.13	0.04
IIGP1	1.08	0.05	1.04	0.01	0.90	0.02
MVP	0.87	0.03	1.19	0.11	1.29	0.07
NAPRT1	0.86	0.04	1.03	0.02	1.11	0.07
NUDT7	2.02	0.39	0.76	0.10	0.78	0.14
PKLR	1.07	0.07	0.87	0.08	0.75	0.02
PPA1	0.82	0.02	1.09	0.03	1.19	0.06
PSMA2	0.91	0.04	1.23	0.07	1.22	0.07
PSMA7	0.90	0.05	1.15	0.03	0.99	0.02
PTGR2	0.89	0.02	1.09	0.07	1.10	0.04
PYGB	1.01	0.05	0.94	0.04	0.80	0.01
RPL4	1.05	0.02	1.00	0.02	0.88	0.01
RPL5	1.05	0.02	0.89	0.04	0.98	0.02
RPLP2	1.10	0.03	0.95	0.03	0.94	0.04
RPS23	1.16	0.06	0.91	0.05	0.89	0.03
RPS5	1.05	0.03	0.91	0.03	0.90	0.03
RTN4	0.88	0.07	1.43	0.09	1.24	0.14
SCPEP1	0.90	0.03	1.46	0.15	1.46	0.15
SERPINA3G	1.27	0.12	0.73	0.08	0.76	0.04

SH3BGRL	0.84	0.04	1.21	0.06	0.92	0.05
SLC25A10	0.86	0.07	1.19	0.05	1.26	0.11
STT3A	1.08	0.04	0.98	0.03	0.86	0.05
SULT1A1	0.82	0.05	1.25	0.07	1.30	0.11
TLN1	0.92	0.03	1.06	0.02	1.08	0.03
TTC38	0.98	0.04	1.10	0.04	1.18	0.05
UOX	1.08	0.06	0.93	0.07	0.79	0.01
VAT1	0.79	0.10	1.05	0.04	1.47	0.10
YBX1	1.12	0.09	0.74	0.03	0.99	0.05

Supplementary Table 12. Details of the liver samples from the rapamycin study used for proteomics analysis.

**Genotype:**	**WT**	**WT**	**WT**	**WT**	**WT**	**WT**	**WT**	**WT**
**Strain:**	**C57BL/6NJ**	**C57BL/6NJ**	**C57BL/6NJ**	**C57BL/6NJ**	**C57BL/6NJ**	**C57BL/6NJ**	**C57BL/6NJ**	**C57BL/6NJ**
**Sex:**	**Male**	**Male**	**Female**	**Female**	**Male**	**Male**	**Female**	**Female**
**Diet:**	**Control***	**Rapamycin**	**Control**	**Rapamycin**	**Control**	**Rapamycin**	**Control**	**Rapamycin**
**Dose (ppm):**	0	42	0	14	0	42	0	14
**Duration:**	8	8	8	8	16	16	16	16
**Age[$] (mths):**								
**24**			6	6				
**26**	6	8					8	7
**28**					8	7		

*prepared diet including encapsulation material
[$]Age when mice were euthanized to collect tissue for proteomic analys.

Supplementary Table 13. The table delineates the number of liver samples from the rapamycin study used for proteomics analysis. Identified features are the number of mass spectrometric signals that passed occupancy and outlier filtering and were associated with a peptide and protein. The results of a Student's unpaired, equal variance t-test analysis between control and rapamycin treated individuals is shown at varying statistical stringencies. Minimum significance cutoff for a false discovery rate of <5% is shown in red.

	**Total all groups**	**male 8 wks**	**male 16 wks**	**Female 8 wks**	**Female 16 wks**
**# livers**	56	14	15	12	15
**Identified features**		30,963	34,730	30,234	30,368
**Quantified proteins**	1,641	1,581	1,561	1,500	1,500
**Student's t-test:**					
**p<0.05**		127	164	101	137
**p<0.01**		26	62	53	36
**p<0.001**		**7**	**18**	**14**	**2**
**p<0.0001**		2	3	3	0

Supplementary Table 14. The biological age calculator uses a rudimentary linear model that weighted each of the 14 proteins equally. The table shows the relative expression level and linear correlation coefficient in reference mice to for the panel of 14 selected proteins in the biological age calculator.

	Months of age			
	16	24	32	r^2
ACSL1	1.09	0.91	0.81	0.973
AOX3	1.1	0.89	0.66	0.999
CA3	1.12	0.84	0.56	0.999
CBS	0.97	0.98	1.22	0.78
CES3G	1.04	0.98	0.79	0.917
CROT	1.05	0.93	0.82	0.999
CYP2C44	0.87	1.07	1.27	0.999
ECH1	1.15	0.97	0.84	0.991
ECI1	1.09	0.88	0.8	0.937
GGCX	0.96	1.23	1.17	0.549
GLO1	1.12	0.98	0.72	0.971
POR	0.96	1.07	1.2	0.998
SDS	0.87	1.02	1.44	0.93
SULT1A1	0.97	1.14	1.46	0.97

Supplementary Table 15. Kruskal-Wallis and Jonckheere-Terprstra significance trend test for the panel of 14 selected proteins in male inbred mouse livers.

	Kruskal-Wallis test	Jonckheere-Terprstra Test
	p-value	
Acsl1	9.40E-04	6.10E-05
Aox3	9.10E-05	2.40E-07
Ca3	4.30E-05	4.84E-07
CBS	5.37E-04	2.02E-05
CES3G	9.76E-04	1.37E-05
CROT	3.86E-03	5.70E-05
CYP2C44	6.82E-03	1.63E-03
ECH1	3.10E-03	1.00E-03
ECI1	2.03E-03	4.93E-04
GGCX	5.43E-03	3.36E-04
GLO1	4.54E-04	2.13E-04
POR	1.30E-04	2.56E-07
SDS	1.21E-03	1.63E-03
SULT1A1	2.17E-05	3.50E-08
